# Interspecific competition affects the expression of personality-traits in natural populations

**DOI:** 10.1038/s41598-019-47694-4

**Published:** 2019-08-01

**Authors:** Lucas A. Wauters, Maria Vittoria Mazzamuto, Francesca Santicchia, Stefan Van Dongen, Damiano G. Preatoni, Adriano Martinoli

**Affiliations:** 1 Environment Analysis and Management Unit, Guido Tosi Research Group, Department of Theoretical and Applied Sciences, University of Insubria, Varese, Italy; 2 Department of Biology, University of Antwerp, Antwerp, Belgium

**Keywords:** Behavioural ecology, Animal behaviour

## Abstract

Competition between animal species can cause niche partitioning and shape an individual’s phenotype, including its behaviour. However, little is known about effects of interspecific competition on personality, the among-individual variation in behaviour that is consistent across different spatial and temporal contexts. We investigated whether alien grey squirrels (*Sciurus carolinensis*) influenced the expression of personality traits in native red squirrels (*Sciurus vulgaris*). In Italy, alien grey squirrels replaced native reds through competition for food resources and space, reducing breeding and recruitment in the native species. We compared personality of red squirrels in red-only (no interspecific competition) and red-grey (with interspecific competition) sites, using arena-tests. The trait activity was measured by Open Field Test while sociability and avoidance were quantified by Mirror Image Stimulation test. Red squirrels co-occurring with the alien species had higher sociability scores and higher between-individual variation in sociability than in red-only sites. Differences in activity and avoidance were not significant. Personality – fitness relationships were not affected by presence or absence of grey squirrels, suggesting that the expression of sociability in red squirrels was not due to short-term selection, but was likely the result of context-related advantages when co-occurring with the competing species.

## Introduction

Intraspecific competition among individuals in a population can be an important driver of natural selection^1,2^. Those individuals that are best adapted to local conditions, through their genotype, morphology, physiology and/or behaviour, will achieve a higher fitness. Not only intrinsic factors affect the outcome of competition, but spatio-temporal variation in extrinsic environmental conditions can produce extra selective pressures that differ among populations and with time. One of these extrinsic factors is the intensity of interspecific competition with a species that occupies an overlapping ecological niche^3–5^. Interspecific competition for limited resources (food, nest sites) can exert selective pressures on all aspects of an animal’s phenotype, including its behaviour^4,6,7^.

Since many behaviours have both a heritable and a flexible component, and their costs and benefits in terms of fitness will vary with environmental changes, the maintenance of behavioural variation can be explained by an evolutionary stable strategy or by a conditional strategy (e.g.^8,9^). In a conditional strategy, the behavioural tactic an individual will adopt depends on some aspect of its environmental or physiological state^8^. Hence the absence or occurrence of competitors belonging to another species, presenting a change in the environmental state, might induce changes in the behaviour of the target species and behaviours less adapted in a single-species situation might become more adaptive when co-occurring with one or more competing species^4,7,10,11^.

Together with a suite of flexible behaviours, animals also display behaviours that differ consistently between individuals across different spatial and temporal contexts, referred to as personality traits^12,13^. Where it has been shown that spatio-temporal variation in the intensity of intraspecific competition (e.g. differences in population density, food availability, habitat use) can affect the relationship between an animal’s personality and its fitness^14–16^, only few studies that considered also a possible relationships between interspecific competition and the expression of personality traits^3,11,17,18^.

A special case of interspecific competition can occur as the result of human-induced biological invasions^7^. Understanding how animals respond to the occurrence of alien (invasive) species, is a critical ecological and evolutionary issue: behavioural responses can play an important role in the interactions between the native and the alien species and certain personality types might be better adapted than others to cope with the new challenge^7^.

In this study we explore whether the occurrence of a competing alien species results in changes in personality traits in comparison to a “single species” situation. We use the well-known study system of interspecific competition between the introduced invasive Eastern grey squirrel (*Sciurus carolinensis*) and the native Eurasian red squirrel (*Sciurus vulgaris*)^19–21^. Although, eventually, competition between the two species results in the replacement of red by alien grey squirrels (e.g.^20,21^; but see^22^), the earlier phases of colonization by the alien species and the years of co-occurrence of both species allow us to test the predictions of the hypothesis that interspecific competition influences the adaptiveness and hence the relative occurrence of different personality traits in the target species. Grey squirrels compete with native reds for limited food resources (tree seeds) and for space to establish home ranges, reducing recruitment of individuals of the native species^20,23,24^. Under the scenario of interspecific competition, more sociable red squirrels should be better adapted to persist the increasing pressure from co-occurring grey squirrels than individuals that tend to avoid close proximity of other squirrels, in particular when dispersal could be personality-trait (avoidance) dependent^7^. Moreover, active, exploring individuals of the native species should be more likely to acquire sufficient food resources, despite the interspecific home range overlap^19,25–27^. Therefore, we expect more explorative and/or active red squirrels and more individuals with a high score for sociability in areas where the native species has to compete with the invader, than in areas with only red squirrels. To test these predictions, we measured personality traits in six populations of the Eurasian red squirrel, three in areas where only the native species occurs and three in areas with both red and grey squirrels. Details of study sites and arena tests are given in the methods and in the Supplementary Material (Table S1 and Section S2).

## Results

### Expert-based personality traits

During OFT, red squirrels spent most time in behaviours related to activity and shyness and little time in exploration. Sociability and avoidance were the most commonly expressed personality traits during MIS (Tables 1 and 2). We did not record any event of attack towards the mirror. Activity and shyness had high repeatability, but repeatability of exploration was very low and so was time spent exploring the arena (Tables 1 and 2). During MIS, the personality traits sociability, avoidance and other had moderate repeatability, while alert had not (Table 2).

**Table 1:** Ethogram for Open Field and Mirror-Image Stimulation tests.

Open Field Test			Mirror Image sStimulation Test		
Behaviour	Behaviour description	Personality trait	Behaviour	Behaviour description	Personality trait
Locomotion	Jump, walk	**Activsity**	Locomotion	Jump, walk	**Other**
Rise	Rise up on hind legs		Rise	Rise up on hind legs	
Scan	Head moving		Scan	Head moving	
Scratch	Scratch or chew floors/walls	**Exploration**	Scratch	Scratch or chew floors/walls	
Sniff	Sniff the corner of arena		Sniff	Sniff the corner of arena	
Head dip	Put head in holes in the floor		Head dip	Put head in holes in the floor	
Hang	Hang on walls	**Shyness**	Hang	Hang on walls	**Avoidance**
Immobile	No movement		Back	Immobile in back half of arena furthest from mirror	
			Slow	Slow approach towards mirror, with hind legs stretched out behind	**Sociability**
			No-aggressive	Non aggressive contact with the mirror	
			Front	Immobile in front half of arena closest to mirror	
			Watch	Immobile, watching directly to mirror	**Alert**
			Attack	Strike the mirror with front legs or head	**Aggressiveness**

Description of the single behaviours and indication of the expert-based grouping into categories that represent personality traits^40^.

**Table 2:** The average proportion of time (raw data) red squirrels were engaged in behaviours related to the different personality traits defined by the expert-based approach during OFT and MIS.

		red-only (n = 156)		red-grey (n = 167)		Repeatability (n = 230)		
Personality trait		Mean	SD	Mean	SD	R	95% CI	Posterior mode
OFT	Activity	0.36	0.19	0.33	0.18	0.50	0.36–0.63	0.50
	Shyness	0.55	0.22	0.57	0.21	0.52	0.39–0.65	0.53
	Exploration	0.06	0.05	0.06	0.07	0.09	0.02–0.18	0.09
MIS	Sociability	0.12	0.26	0.26	0.33	0.19	0.05–0.33	0.18
	Avoidance	0.57	0.31	0.47	0.35	0.20	0.07–0.33	0.18
	Alert	0.12	0.10	0.12	0.13	0.09	0.01–0.17	0.07
	Other	0.17	0.17	0.13	0.13	0.34	0.20–0.49	0.35
	Aggressiveness	0 attacks to the mirror						

Data grouped by situation (study sites with only red squirrels = red-only; study sites with both red and grey squirrels = red-grey). Repeatability estimated with the MCMCglmm model (see methods).

### Interspecific competition and personality

**Figure 1:**
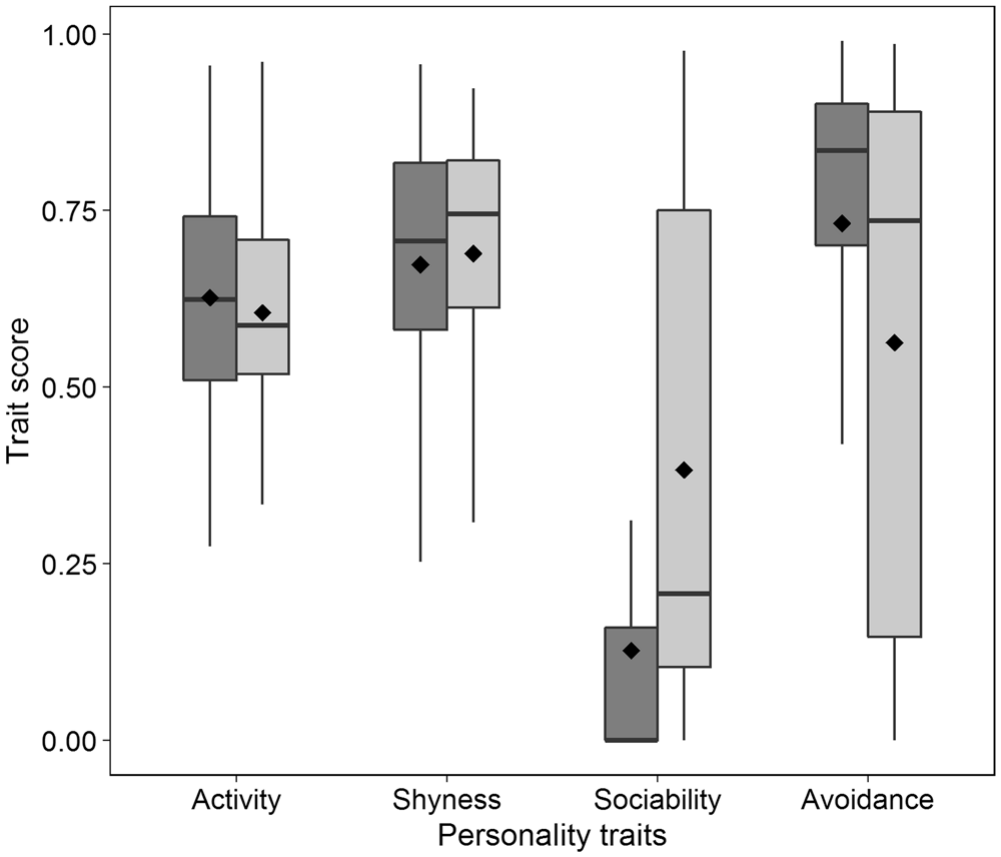
Box and Whisker plots of the personality trait scores (squareroot transformed proportion of time spent in behaviours that are part of the given trait) for the four main traits. Comparison between red-only (dark grey) and red-grey (light grey) area-type. Diamonds = mean. Data shown using the first arena test for each individual (n = 184; 95 from red-only area type and 89 from red-grey area type).

Since exploration had low repeatability and its average score did not differ between red-only and red-grey sites (Table 2), we will only report the traits activity and shyness from OFT. There was no effect of the presence of grey squirrels on the expression of activity (estimate β = −0.08, 95% CI = −0.45 to 0.26, pMCMC = 0.59) or on the expression of shyness (β = 0.06, 95% CI = −0.29 to 0.39, pMCMC = 0.71) (Fig. 1). Red squirrels tended to be more active during OFT in 2017 than in 2016 (β = 0.32, 95% CI = 0.08 to 0.56, pMCMC = 0.009) and scores for activity were highest in the first test, while those for shyness were lowest in the first test (for estimates see Supporting Information, Table S3). Hence, when red squirrels were in the arena for the first they performed more activity-related behaviours than in subsequent tests.

Patterns of sociability measured by MIS differed between red-only and red-grey sites (Fig. 1). Behaviours related to sociability were more expressed in red-grey than in red-only situation (β = 0.44, 95% CI = 0.06 to 0.83, pMCMC = 0.034). Moreover, red squirrels tended to express slightly more avoidance in red-only than in red-grey situation (β = 0.17, 95% CI = −0.16 to 0.49, pMCMC = 0.29), but this difference was not significant. Individual variation in personality was not affected by sex or body mass (Supporting Information, Table S3), except for a sex-effect on the trait avoidance: females expressed less avoidance than males (β = −0.29, 95% CI = −0.55 to −0.02, pMCMC = 0.033). The correlations between personality traits are reported in Supplemental Information (Table S4).

### Relationships of personality with local survival and reproduction

We estimated local survival on 224 observations (112 red-only sites; 112 red-grey sites) of 180 different red squirrels. Overall, in 71% of cases red squirrels survived the year (73% in red-only sites, 70% in red-grey sites). There was no effect of area-type (red-grey vs red-only) on probability to survive (β = −14.8, 95% CI = −53.2 to 19.1, pMCMC = 0.31). Personality traits were not related with the probability of survival (Table 3) and neither was body mass (β = 4.89, CI = −9.46 to 19.4, pMCMC = 0.38).

**Table 3:** Correlation coefficients and 95% credibility intervals derived from the MCMCglmm models between the two fitness components (probability of local survival and probability to produce a litter) and four personality traits of red squirrels.

Study area type	Fitness variables	Activity	Shyness	Sociability	Avoidance
All	Local survival	−0.03 (−0.28–0.23)	0.03 (−0.21–0.28)	−0.25 (−0.79–0.27)	0.13 (−0.35–0.64)
	Female reproduction	0.29 (−0.11–0.77)	−0.34 (−0.73–0.02)	0.24 (−0.63–0.92)	−0.32 (−0.91–0.37)
Red-only	Local survival	−0.09 (−0.47–0.29)	0.18 (−0.20–0.55)	−0.38 (−0.96–0.37)	0.19 (−0.52–0.89)
	Female reproduction	0.46 (−0.13–0.98)	−0.53 (−0.99–0.02)	0.30 (−0.45–0.92)	−0.30 (−0.93–0.36)
Red-grey	Local survival	−0.12 (−0.49–0.24)	0.05 (−0.28–0.39)	−0.06 (−0.48–0.36)	0.04 (−0.37–0.43)
	Female reproduction	−0.09 (−0.62–0.41)	0.07 (−0.40––0.57)	−0.30 (−0.77–0.23)	0.26 (−0.28–0.73)
Difference slopes	Local survival	−0.03 (−0.56–0.52)	−0.13 (−0.64–0.38)	0.31 (−0.62–1.03)	−0.16 (−0.94–0.72)
	Female reproduction	−0.55 (−1.41–0.45)	0.60 (−0.35–1.45)	−0.60 (−1.38–0.31)	0.57 (−0.37–1.37)

All correlations include 0 in the 95% CI. Differences between posterior slopes of the correlation estimates for red-only and red-grey area-type.

We estimated the probability to reproduce using 77 observations (32 red-only sites, 45 red-grey sites). In 62% of cases female red squirrels produced a litter (59% in red-only sites, 64% in red-grey). The probability to produce at least one litter per year did not differ between red-grey and red-only sites (β = −27.9, 95% CI = −83.2 to 17.8, pMCMC = 0.16). Reproductive output was lower in 2017 than in 2016 (β = −48.1, 95% CI = −91.3 to −20.3, pMCMC < 0.001) and increased with a female’s body mass (β = 43.5, 95% CI = 15.1 to 81.9, pMCMC < 0.0001). The probability to reproduce was not related with any of the personality traits (Table 3).

Next, we run two sub-models, one for each area-type, and compared their posterior slopes for the various correlations between personality traits and fitness components to explore whether high levels of activity and/or sociability had a fitness advantage in red-grey sites, but not in red-only. Correlations with survival or reproductive rate were weak for all traits reported and in both red-only and red-grey sites, and there was no significance difference in the posterior slopes for any of the traits (Table 3; Supporting Information, Table S5). Hence, there was no evidence for selection favouring active or social red squirrels in populations co-occurring with the alien competitor. However, the between-individual variance in sociability and avoidance of red squirrels was higher when the competitor was present than in red-only sites (Table 4).

**Table 4:** Mean (95% CI) between-individual variances and differences (Diff) in between-individual variances of the personality traits in the Red-grey and Red-only areas (Differences Red-grey – Red-Ony) based on the two MCMCglmm models (iterations 1000000, burnin 50000, thinning interval 40, sample size per chain 25000).

Personality trait	Red-Grey areas	Red-Only areas	Diff. Mean ± SD	Diff. 95% CI
Activity	0.33 (0.13–0.56)	0.49 (0.21–0.80)	−0.16 ± 0.19	−0.54 to 0.20
Exploration	0.11 (0.01–0.23)	0.24 (0.05–0.45)	−0.13 ± 0.12	−0.40 to 0.09
Shyness	0.45 (0.21–0.71)	0.53 (0.21–0.88)	−0.08 ± 0.22	−0.52 to 0.34
Sociability	0.42 (0.11–0.73)	0.06 (0.01–0.14)	0.35 ± 0.17	0.06 to 0.72
Avoidance	0.43 (0.16–0.74)	0.10 (0.01–0.21)	0.33 ± 0.16	0.05 to 0.68
Alert	0.10 (0.01–0.23)	0.11 (0.01–0.25)	−0.01 ± 0.09	−0.20 to 0.18
Other	0.30 (0.10–0.52)	0.45 (0.15–0.77)	−0.15 ± 0.19	−0.55 to 0.22

## Discussion

In small mammals personality is studied using capture-mark-recapture data and/or by arena tests^15,28,29^. Using arena test (Open Field Test followed by a Mirror Image Stimulation test) we found that red squirrels co-occurring with grey squirrels expressed more the personality trait sociability than in areas without the invasive competitor. The tendency to show less avoidance of the mirror image in red-grey than in red-only areas was weak and non significant, and in contrast with our predictions, we found no difference in the expression of activity between red-grey and red-only areas. The between-individual variation in the traits sociability and avoidance among red squirrels was higher in sites with the alien competitor than in red-only sites. This indicates more variation among individual red squirrels in the expression of these traits in the situation with interspecific competition (see also Fig. 1).

There are two, non mutually exclusive explanations for the pattern of sociability expression. The first implies interspecific competition as a driver for natural selection favouring certain phenotypes (personality traits) over others (character displacement, e.g.^3,4,6^); the second that the observed differences are the results of context related (with vs without competitor) plasticity in the behaviour of red squirrel.

In the first case, interspecific competition between grey and red squirrels favours those individuals of the native species that have a higher sociability, measured by the reactions to their mirror image. In other words, red squirrels that behave in a sociable, non-aggressive way to conspecifics are more common in red-grey than in red-only areas because they have a selective advantage. This advantage could be related to a general social personality trait, implying non-aggressive behaviour and tolerance to close proximity not only to a conspecific, measured with MIS, but also to the competing invasive species^26^. If these personality trait differences are the result of selection, we expect that red squirrels with a higher sociability score have a survival and/or reproduction benefit in the woodlands with grey squirrels, but not in woodlands without the competitor. We expected positive correlations of sociability with one or both fitness components only in red-grey sites, but no significant associations were found. The same was true for the trait avoidance in red-only sites. Hence, our results suggested that the observed differences in the expression of personality traits by red squirrels in red-only than in red-grey study sites were not due to selection on these traits (sociability and avoidance). It must be noted that differences in personality among animal can also affect their dispersal behaviour^7,30^. Since we were unable to follow juvenile cohorts throughout the dispersal and settlement process^31^, we could not rule out that selection for higher sociability occurred in red squirrels when co-existing with grey squirrels during this phase. Studying the personality of juvenile and subadult red squirrels and its relationship with dispersal and settlement success can reveal possible selection mechanisms for certain traits that might differ between areas with and without grey squirrels.

One important limitation of our dataset is that the proxy of fitness did not account for variation in number of young weaned/female as a component affecting variation in reproductive success^32^. Moreover, our study may have been too short (2 years) to measure any selective advantage of a given personality trait (see also^18^). Despite these potential problems, we argue that it is unlikely that competition with grey squirrels asserts a selective pressure on co-occurring native red squirrels in our study system. Grey squirrels colonized our study sites very recently, between 2 and 8 years before the arena test experiments. Hence, selection on personality traits should have occurred in only two-three generations, which seems unlikely (but see^33^). Also, keeping grey squirrel densities low by removal could decrease the intensity of interspecific competition resulting in reduced selective pressure on local adaptations of personality. Finally, other phenotypic characteristics may have more pronounced effects on fitness than personality traits; in fact we found strong positive effects of a squirrel’s body mass on reproduction, in agreement with earlier studies^16,32,34^.

The second explanation of having more red squirrels with high sociability in sites with grey squirrels is that sociability has a marked flexible component itself, or is related with other behaviours that have context-related plasticity and facilitate red squirrels to share the woodland with the invasive competitor. In woodlands occupied by both species, the interspecific overlap of the foraging-niche, daily activity pattern and home ranges (core-areas) are high^19,26^ and more sociable red squirrels are likely to sustain such pressure, that increase with grey squirrel density, better than individuals with a tendency to avoid conspecifics. Also, higher sociability could be related with a lower susceptibility to physiological stress induced by the invader^35^. Conversely, dispersal as a conditional strategy^36^, could result in red squirrels with a strong avoidance personality being the first to emigrate from woodlands invaded by grey squirrels, as supported also by low local recruitment rates of juvenile red squirrels in areas of co-occurrence^20^. Finally, Sih *et al*.^7^ reported that personality traits can influence the intensity of interspecific interactions and/or increase intraspecific variation of certain traits, which might result in higher functional diversity for one or both of the competing species. Our data supported this hypothesis, since we found higher between-individual variance in sociability among red squirrels co-occurring with grey squirrels than among red squirrels in red-only sites.

As far as the relationship between interspecific competition and activity was concerned, we predicted more active squirrels in areas where the native species has to compete with the invader, since higher activity was thought to be related with greater food resource acquisition. However, we did not find any differences in the activity trait comparing the two situations (red-only vs red-grey). We believe this was due to our grey squirrel control to keep their densities low. At such low densities, interspecific competition for food is reduced and might be insufficient to create a marked advantage for more active red squirrels.

The study was carried out in six study sites that were not identical in tree species composition or red squirrel density (see also study design below). This was addressed statistically by modelling study site nested within situation as a random effect in the MCMCglmm, thereby correcting for any potential between site variation in the test for the situation effect (red-only vs red-grey situation). Since we did find a significant effect of situation on sociability expression, this effect was much larger than any potential between study site variation. In other words, any variation in the expression of personality traits, potentially due to differences between study sites in the proportions of conifers and deciduous tree species, or other ecological variables, was much smaller than the effect of the presence of grey squirrels on the expression of sociability.

Few studies investigated individual differences in personality in relation to outcomes of interspecific competition. Experiments with two ecologically similar fish species, the threespine and ninespine sticklebacks (*Gasterosteus aculeatus* and *Pungitius pungitius*) showed that more active individuals of both species spend more time in open waters than in vegetation, and bolder fish had a higher prey-consumption rate than more shy individuals, irrespective of species^17^. Authors suggested that individual variation in personality traits can facilitate interspecific niche overlap, which might affect prevailing selection pressures in areas where interspecific competition is more important compared to single-species situations (see also^3,11^). In birds, territorial aggression can be very important in the context of interspecific competition for limited high-quality nesting sites^18^. Eastern bluebirds (*Sialia sialis*) showed a strong tendency toward assortative mating in areas of both high and low interspecific competition with tree swallows (*Tachycineta bicolor*), but pairs that behaved the most similarly and displayed either extremely aggressive or extremely non-aggressive phenotypes experienced higher reproductive success only in areas of high interspecific competition^18^. However, since the study was over a single breeding season, they could not measure ongoing selection of bluebird personality traits driven by interspecific competition. These authors suggested that interspecific competition may select for certain personality traits and that animal personality may be an important factor influencing the outcome of interactions between native and invasive species^18^.

In conclusion, our data showed that, of the different personality traits investigated, only the sociability of red squirrels changed in sites invaded by grey squirrels. Red squirrels competing with the invasive species had higher sociability scores and higher between-individual variance in sociability than in sites without grey squirrels. Although it was recently shown that natural selection of personality traits and emergence of behavioural syndromes can be rapid^33^, we found no evidence that the observed differences in personality traits were the consequence of character displacement driven by interspecific competition. However, differences in dispersal tendency of individual red squirrels that are either social or avoiders could explain the higher average scores of sociability in woods shared with grey squirrels than in woods without the invasive competitor. Further studies over a longer time-period should investigate whether the flexible component of the activity, sociability and avoidance personality traits vary over time with the increasing experience of the individual squirrel. Moreover, allowing grey squirrel density to increase in some study sites might reveal whether interspecific competition can drive selection for personality phenotypes that allow red squirrels to cope with the alien invasive species. More research on naturally co-occurring species in a guild and how both intra- and interspecific interactions contribute to the selection of personality traits is mandatory to increase our insight in the role of interspecific competition in shaping individual variation in personality.

## Methods

### Study design and trapping squirrels

The six red squirrel populations (study sites) we monitored are independent replicates in the same geographic area (North Italy): three with only red squirrels and three with both red and grey squirrels. Since we used a natural setting, the six study sites were not identical in forest composition or red squirrel density. However, the range of densities was comparable between red-only and red-grey sites (Table S1) and social organisation, mating behaviour, foraging behaviour and activity patterns are similar and consistent over a wide range of habitat types^23,25,26,37^. Therefore, there should be no confounding ecological variables associated with the different study sites that could influence the main effect of absence/presence of the alien competitor. Moreover, site heterogeneity was addressed statistically by adding study site nested in area-type as random effect in the MCMCglmm model (see statistical analyses). The red-grey sites are mature mixed broadleaf-conifer woods dominated by oaks (*Quercus robur*, *Q. petraea*) and hornbeam (*Carpinus betulus*) with different proportion of conifers. The red-only sites are mixed conifer forests and data on forest structure and composition are reported elsewhere^37,38^.

Trapping was carried out in two to four periods per year between January 2016 and December 2017 (Supporting Information, Table S1). A trapping session involved the use of Tomahawk “squirrel” traps (models 201 and 202, Tomahawk Live Trap, WI, USA) placed on the ground or at breast height against tree trunks. Traps were more or less homogeneously distributed over the study area, with average trap densities varying among sites, in relation to expected squirrel density (Supporting Information, Table S1).

Traps were pre-baited with sunflower seeds and hazelnuts 4 to 6 times over a 30 day period, then baited and set for 4–5 days. Traps were checked two times per day. Each trapped red squirrel was flushed into a light cotton handling bag with a zipper or a wire-mesh “handling cone” to minimize stress during handling, and individually marked using numbered metal ear-tags (type 1003 S, National Band and Tag, Newport, KY, USA). It was weighed to the nearest 5 g using a spring-balance (Pesola AG, Baar, Switzerland). Sex, age class and reproductive condition were determined on the basis of external genitalia, condition of the nipples (females) and body mass, with juvenile red squirrels weighing less than 250 g^32^.

We used capture-mark-recapture (CMR) data to define local annual survival (binary variable: 1 = survived, trapped from first to last trapping session in a given year; 0 = not survived, no longer trapped in the last trapping session of the given year, nor in subsequent sessions). Capture probabilities in red squirrel populations are high, and both bold and shy animals are trapped at least once per year; moreover, radio-tracking data confirm survival estimates based on CMR^16^. For females we also determined a measure of reproductive output: each individual female was scored 1 (binary variable) when it produced a litter (trapped pregnant and/or lactating in at least one session), it was scored 0 when no litter was produced (anoestrus and non lactating in all trapping sessions in a given year).

In the experimental sites, captured grey squirrels were removed as part of a red squirrel conservation project: animals were euthanized by CO_2_ inhalation, following the EC and AVMA guidelines^39^. Doing so, grey squirrel densities were kept low, making any result of the relationships between interspecific competition and red squirrel personality conservative. Trapping and handling squirrels complied with current laws on animal research and welfare in Italy.

### Ethical approval for fieldwork with animals

Trapping, marking and handling of red squirrels and arena-test experiments were carried out in accordance with the Guidelines for the Use of Animals in Research (Animal Behaviour, 2018, 135, I-X). Grey squirrel control was carried out in accordance to the indications in Leary S. *et al*. 2013 AVMA Guidelines for the Euthanasia of Animals: 2013 Edition. Approval and legal requirements according to the Italian Wildlife Protection and Hunting Law L.N. 157 from 1992 and authorizations N.294–34626 of 12/09/2014 (2014–2016) from the Provincia di Torino and N62-3025 (2017–2019) from the Città Metropolitana di Torino, and Decreto N. 11190 (29/11/2013) and decrees n°9523 of 15/10/2014 and n° 198 (13/01/2017) from Direzione Generale Agricoltura, Regione Lombardia; and the permission Protocol n° 414 of 28/02/2014 of the Stelvio National Park.

### Measuring personality

Details of arena tests in Supplementary material 2 (and see^40^). To quantify individual personality, we performed two different experiments inside the arena: Open Field Test (OFT) to estimate activity and exploration levels in a novel environment and Mirror Image Stimulation (MIS) to test aggressiveness, sociability or avoidance towards conspecifics^28,40–42^. The two tests were performed in the same testing session, with the OFT also serving as habituation time before the MIS. We performed arena tests for each individual only once per capture-session to reduce stress and habituation in animals (minimum time between tests for the same individual: 77 days). In addition, to check the assumptions of repeatability of personality traits we repeated both experiments (OFT and MIS) in different capture-sessions to have at least two arena tests for most individuals.

In total we performed 323 arena tests (156 in red-only sites, 167 in red-grey sites) on 184 different red squirrels (95 in red-only sites, 89 in red-grey sites; Table S1). We analysed digital videos of OFT and MIS with CowLog 3.0.2 software^43^ and used the ethogram from Mazzamuto *et al*.^40^ (Table 1); for each experiment, the software calculates the time that an individual spent in each behaviour.

### Statistical analysis

We first transformed the time calculated by CowLog 3.0.2 in proportion of time spent by each squirrel in a given behavioural state. To reduce the number of behaviours observed into few personality-linked variables we used the expert-based method described previously^40^. With the expert-based approach the researcher defines groups of behaviours, with each group related to a specific personality trait, summing the values of the single behaviours to obtain scores for each personality trait^40^. The method was validated by comparing its performance of grouping behaviours into personality traits with the outcomes of statistical grouping based on PCA or Factor Analysis^40^. Aggressiveness was considered as the number of attacks towards the mirror during MIS.

All analyses and interpretations were based on a multivariate mixed model fitted in a Bayesian framework using the package MCMCglmm in R^44^. Personality-trait scores were squareroot transformed before analysis. All expert-based personality traits, survival and reproduction were treated as dependent variables after standardisation. For all expert-based personality traits, a Gaussian residual error distribution was used, while survival and reproduction were treated as binomial. Assumption of multivariate normality of the personality traits was supported by the QQ-plot of the Mahalanobis distances of the model residuals (r-squared value = 0.92). As repeated observations were present, individual was added as a random effect. Because 91 individuals (60 males, 31 females) were caught in at least two trapping sessions (a total sample of 230 tests), we were able to estimate the repeatability of the expert-based personality traits as the between-individual variation divided by the sum of the between-individual and residual variation. For both the residual and between-individual variation, an unstructured variance-covariance matrix was modelled, allowing the estimation of correlations among the response variables (covariance divided by the square root of the product of the variances). Area-type, red-only vs red-grey, was treated as fixed effect, and area nested within area-type was added as random effect (as a heterogeneous identity matrix) to avoid pseudoreplication problems during the parameter estimation process. In addition, sex, body mass, year and arena test order (first to fourth test of the same animal) were added as fixed effects. We did not include body mass measures of pregnant females to avoid a bias due to extra weight of developing embryos. The effect of sex was set to zero for the dependent variable reproduction and the effect of arena test order was set to zero for both reproduction and survival. Posterior distributions were based on 10000000 iterations with a burnin of 50000 iterations and thinning of 100, such that 100000 iterations were used to obtain point estimates and 95% credibility intervals (model with 1000000 iterations, 50000 burnin and 40 thinning produced the same results). For all fixed effects, the prior distribution was Gaussian with zero mean and variance equal to 1. For the random effects and residual variation and inverse Wishard prior was set with diagonal elements equal to 0.5, 0.5 and 0.1 for the residual, between-individual and nested area effect respectively. The believe parameter was set to 0.01. Full model outputs are provided in Supporting Information, Table S3.

To explore whether high levels of activity and/or sociability had a fitness advantage in red-grey sites but not in red-only sites, we ran sub-models, one for each area-type. These models were constructed as the full model except for the fixed effect of area-type (full outputs in Supporting Information, Table S5). We then tested explicitly for the interactions with area-type by comparing the slopes of the posterior distributions from the two separate models, for the various correlations between personality traits and fitness components (survival and reproduction).

## Supplementary information

Supplemental Material Wauters et al. Interspecific competition affects the expression of personality-traits in natural populations

## Data Availability

MCMCglmm outputs are available in the Supplemental Information. The MCMCglmm and other data analyses R-scripts and the datafile are available at 10.5281/zenodo.1451460.
