# Supplementary material for: Interspecific competition affects the expression of personality-traits in natural populations
Source: Sci Rep. 2019 Aug 1;9:11189. doi: 10.1038/s41598-019-47694-4 (PMC6673699; doi:10.1038/s41598-019-47694-4)
Supplement: Supplementary file 1 — Supplemental Material Wauters et al. Interspecific competition affects the expression of personality-traits in natural populations [file 41598_2019_47694_MOESM1_ESM.pdf]

Lucas A. Wauters *[a, b], Maria Vittoria Mazzamuto * [a], Francesca Santicchia [a], Stefan Van Dongen[b], Damiano G. Preatoni[a], Adriano Martinoli [a]

(* contributed equally to this manuscript)

Supplemental Material

Table S1. Location of study sites in Lombardy and Piedmont, North Italy. Sample size refers to the number of arena tests (total n = 323). In all sites, the density of red squirrels (highest density of different trapping periods) was estimated by MNA, and minimum density of grey squirrels (in red-grey sites) was estimated from removals.

**Site-type (size, ha)**	**Coordinates**	**Sample size (M, F)**	**Red squirrel density (ha^{-1})**	**Grey squirrel density (ha^{-1})**	**N capture sessions (N traps)**	
					2016	2017
**Red-only**						
Valfurva (76 ha)	46°27′N, 10°31′E	84 (60, 24)	0.60	n/a	2 (20)	2 (20)
Bormio (90 ha)	46°27′N, 10°30′E	41 (36, 5)	0.24	n/a	2 (20)	2 (20)
Cancano (54 ha)	46°33′N, 10°15′E	31 (20, 11)	0.13	n/a	2 (18)	2 (18)
**Red-grey**						
Vanzago (74 ha)	45°31′N, 8°58′E	45 (27, 18)	0.28	0.24	3 (27)	4 (28)
Castelbarco (55 ha)	45°35′N, 9°31′E	88 (53, 35)	0.30	0.67	4 (39)	6 (41)
Passatempo (18 ha)	45°00′N, 7°78′E	34 (18, 16)	0.38	0.98	3 (21)	4 (20)

Supplemental material 2. Arena test details.

Immediately after handling, we released a marked squirrel in the arena by opening a sliding door (28 × 15 cm, internal opening 12 x 12 cm) and allowing the animal to move from the handling bag into the arena. The arena is a white extruded polycarbonate box of 50 × 51 × 51 cm; the floor of the arena consists of a panel with four blind holes (7 cm diameter × 4 cm deep), that allow to differentiate between exploration and activity behaviours (hole board test, [41,43]). The wall at the opposite site of the entrance has a sliding panel that can be removed to reveal a mirror (24 × 51 cm). In the lid of the arena (inside a 5 cm diameter hole) we fit a web camera (Drift, Professional HD Action Camera, model: FD9960, Ghost S) to record the animal's behaviour. To quantify individual personality, we performed two different experiments inside the arena: Open Field Test (OFT) to estimate activity and exploration levels in a novel environment and Mirror Image Stimulation (MIS) to test aggressiveness, sociability or

avoidance towards conspecifics [28,42]. The two tests were performed in the same testing session, with the OFT also serving as habituation time before the MIS. The arena was placed on the ground near the trap where the squirrel was caught and recording (OFT experiment) started before we released the animal inside the arena. After 4 min we opened the mirror and began MIS test for another 3 min [41]. At the end of MIS the squirrel was released by opening the sliding door. After each experiment the arena was cleaned with 90% ethyl alcohol to eliminate urine and faecal pellets when present and to eliminate effects of squirrel's scent on behaviour of the next animal.

Table S3. MCMCglmm outputs of the full model (for detailed description see Statistical Analysis). All expert-based personality traits, survival and reproduction were treated as dependent variables after standardisation. For all expert-based personality traits, a Gaussian residual error distribution was used, while survival and reproduction were treated as binomial. Assumption of multivariate normality of the personality traits was supported by the QQ-plot of the Mahalanobis distances of the model residuals (r-squared value = 0.92). For both the residual and between-individual variation, an unstructured variance-covariance matrix was modelled, allowing the estimation of correlations among the response variables (covariance divided by the square root of the product of the variances). Area-type, red-only vs red-grey, was treated as fixed effect, and area nested within area-type was added as random effect (as a heterogeneous identity matrix) to avoid pseudoreplication problems during the parameter estimation process. In addition, sex, body mass, year and arena test order (first to fourth test of the same animal) were added as fixed effects. The effect of sex was set to zero for the dependent variable reproduction and the effect of arena test order was set to zero for both reproduction and survival. Posterior distributions were based on 10000000 iterations with a burnin of 50000 iterations and thinning of 100, such that 100000 iterations were used to obtain point estimates and 95% credibility intervals. For all fixed effects, the prior distribution was Gaussian with zero mean and variance equal to 1. For the random effects and residual variation and inverse Wishard prior was set with diagonal elements equal to 0.5, 0.5 and 0.1 for the residual, between-individual and nested area effect respectively. The believe parameter was set to 0.01.

Model output

```
DIC: 2975.358

G-structure:  ~us(trait):IDf (Among individual variance – covariance estimates)

                             post.mean   l-95% CI   u-95% CI  eff.samp
traitshy:traitshy.IDf         5.120e-01  3.165e-01  7.084e-01 20318.712
traitexp:traitshy.IDf        -1.603e-01 -2.899e-01 -3.648e-02 11947.318
traitact:traitshy.IDf        -4.656e-01 -6.478e-01 -2.910e-01 21410.384
traitsoc:traitshy.IDf        -6.582e-02 -1.901e-01  6.097e-02 13014.822
traitavo:traitshy.IDf         1.691e-01  3.877e-02  3.034e-01 13501.102
traitale:traitshy.IDf        -8.327e-02 -1.988e-01  3.150e-02 16747.175
traitoth:traitshy.IDf        -3.688e-01 -5.302e-01 -2.146e-01 16575.455
traitSURVf.2:traitshy.IDf     1.712e+00 -1.377e+01  1.825e+01   439.269
traitREPRODf.2:traitshy.IDf  -1.238e+01 -3.035e+01  1.825e+00    22.645
traitshy:traitexp.IDf        -1.603e-01 -2.899e-01 -3.648e-02 11947.318
traitexp:traitexp.IDf         9.551e-02  2.023e-02  1.827e-01  3032.369
traitact:traitexp.IDf         1.394e-01  2.863e-02  2.550e-01 16024.791
traitsoc:traitexp.IDf         4.050e-02 -3.210e-02  1.151e-01  1837.460
traitavo:traitexp.IDf        -6.515e-02 -1.444e-01  7.918e-03  3492.492
traitale:traitexp.IDf         2.860e-02 -2.948e-02  8.866e-02  2615.823
traitoth:traitexp.IDf         1.251e-01  2.724e-02  2.316e-01  8579.864
```

```
traitSURVf.2:traitexp.IDf         -5.598e+00 -2.230e+01  7.995e+00   519.585
traitREPRODf.2:traitexp.IDf        7.736e+00 -3.099e+00  2.105e+01    81.962
traitshy:traitact.IDf             -4.656e-01 -6.478e-01 -2.910e-01 21410.384
traitexp:traitact.IDf              1.394e-01  2.863e-02  2.550e-01 16024.791
traitact:traitact.IDf              4.539e-01  2.826e-01  6.379e-01 19816.750
traitsoc:traitact.IDf              5.547e-02 -6.215e-02  1.734e-01 13883.258
traitavo:traitact.IDf             -1.456e-01 -2.705e-01 -2.350e-02 13847.234
traitale:traitact.IDf              8.768e-02 -2.215e-02  1.979e-01 17056.542
traitoth:traitact.IDf              3.467e-01  1.994e-01  4.955e-01 14436.423
traitSURVf.2:traitact.IDf         -1.462e+00 -1.781e+01  1.401e+01   288.076
traitREPRODf.2:traitact.IDf        9.570e+00 -4.317e+00  2.874e+01    19.248
traitshy:traitsoc.IDf             -6.582e-02 -1.901e-01  6.097e-02 13014.822
traitexp:traitsoc.IDf              4.050e-02 -3.210e-02  1.151e-01  1837.460
traitact:traitsoc.IDf              5.547e-02 -6.215e-02  1.734e-01 13883.258
traitsoc:traitsoc.IDf              1.197e-01  1.344e-02  2.520e-01  4148.428
traitavo:traitsoc.IDf             -1.122e-01 -2.423e-01 -7.454e-03  4435.971
traitale:traitsoc.IDf              1.194e-03 -7.328e-02  7.255e-02  2300.088
traitoth:traitsoc.IDf              5.217e-02 -5.388e-02  1.613e-01  8139.756
traitSURVf.2:traitsoc.IDf         -6.805e+00 -2.567e+01  7.210e+00   202.827
traitREPRODf.2:traitsoc.IDf        4.227e+00 -1.326e+01  2.064e+01    32.836
traitshy:traitavo.IDf              1.691e-01  3.877e-02  3.034e-01 13501.102
traitexp:traitavo.IDf             -6.515e-02 -1.444e-01  7.918e-03  3492.492
traitact:traitavo.IDf             -1.456e-01 -2.705e-01 -2.350e-02 13847.234
traitsoc:traitavo.IDf             -1.122e-01 -2.423e-01 -7.454e-03  4435.971
traitavo:traitavo.IDf              1.419e-01  2.464e-02  2.774e-01  5177.352
traitale:traitavo.IDf             -2.378e-02 -9.940e-02  5.229e-02  3613.387
traitoth:traitavo.IDf             -1.302e-01 -2.485e-01 -1.832e-02  9975.742
traitSURVf.2:traitavo.IDf          4.361e+00 -1.039e+01  2.302e+01   277.709
traitREPRODf.2:traitavo.IDf       -5.700e+00 -2.016e+01  8.322e+00    56.072
traitshy:traitale.IDf             -8.327e-02 -1.988e-01  3.150e-02 16747.175
traitexp:traitale.IDf              2.860e-02 -2.948e-02  8.866e-02  2615.823
traitact:traitale.IDf              8.768e-02 -2.215e-02  1.979e-01 17056.542
traitsoc:traitale.IDf              1.194e-03 -7.328e-02  7.255e-02  2300.088
traitavo:traitale.IDf             -2.378e-02 -9.940e-02  5.229e-02  3613.387
traitale:traitale.IDf              8.202e-02  8.382e-03  1.686e-01   724.548
traitoth:traitale.IDf              7.033e-02 -2.459e-02  1.673e-01  7678.923
traitSURVf.2:traitale.IDf          6.623e+00 -8.228e+00  2.814e+01   131.287
traitREPRODf.2:traitale.IDf        6.638e+00 -4.551e+00  1.947e+01    69.884
traitshy:traitoth.IDf             -3.688e-01 -5.302e-01 -2.146e-01 16575.455
traitexp:traitoth.IDf              1.251e-01  2.724e-02  2.316e-01  8579.864
traitact:traitoth.IDf              3.467e-01  1.994e-01  4.955e-01 14436.423
traitsoc:traitoth.IDf              5.217e-02 -5.388e-02  1.613e-01  8139.756
traitavo:traitoth.IDf             -1.302e-01 -2.485e-01 -1.832e-02  9975.742
traitale:traitoth.IDf              7.033e-02 -2.459e-02  1.673e-01  7678.923
traitoth:traitoth.IDf              3.233e-01  1.603e-01  4.952e-01 12971.774
traitSURVf.2:traitoth.IDf         -6.376e+00 -2.584e+01  9.393e+00   110.553
traitREPRODf.2:traitoth.IDf        8.600e+00 -4.801e+00  2.526e+01    31.755
traitshy:traitSURVf.2.IDf          1.712e+00 -1.377e+01  1.825e+01   439.269
traitexp:traitSURVf.2.IDf         -5.598e+00 -2.230e+01  7.995e+00   519.585
traitact:traitSURVf.2.IDf         -1.462e+00 -1.781e+01  1.401e+01   288.076
traitsoc:traitSURVf.2.IDf         -6.805e+00 -2.567e+01  7.210e+00   202.827
traitavo:traitSURVf.2.IDf          4.361e+00 -1.039e+01  2.302e+01   277.709
traitale:traitSURVf.2.IDf          6.623e+00 -8.228e+00  2.814e+01   131.287
traitoth:traitSURVf.2.IDf         -6.376e+00 -2.584e+01  9.393e+00   110.553
traitSURVf.2:traitSURVf.2.IDf      7.827e+03  1.583e+01  1.815e+04     2.673
traitREPRODf.2:traitSURVf.2.IDf    1.093e+03 -3.418e+02  3.119e+03    24.453
traitshy:traitREPRODf.2.IDf       -1.238e+01 -3.035e+01  1.825e+00    22.645
traitexp:traitREPRODf.2.IDf        7.736e+00 -3.099e+00  2.105e+01    81.962
traitact:traitREPRODf.2.IDf        9.570e+00 -4.317e+00  2.874e+01    19.248
traitsoc:traitREPRODf.2.IDf        4.227e+00 -1.326e+01  2.064e+01    32.836
traitavo:traitREPRODf.2.IDf       -5.700e+00 -2.016e+01  8.322e+00    56.072
traitale:traitREPRODf.2.IDf        6.638e+00 -4.551e+00  1.947e+01    69.884
traitoth:traitREPRODf.2.IDf        8.600e+00 -4.801e+00  2.526e+01    31.755
traitSURVf.2:traitREPRODf.2.IDf    1.093e+03 -3.418e+02  3.119e+03    24.453
traitREPRODf.2:traitREPRODf.2.IDf  2.976e+03  9.233e+00  7.131e+03     3.309
```

```
               ~idh(trait):AREA_TYPEf:AREAf

                                 post.mean  l-95% CI  u-95% CI eff.samp
traitshy.AREA_TYPEf:AREAf          0.01578 5.767e-05 6.255e-02  96658.9
traitexp.AREA_TYPEf:AREAf          0.02737 7.669e-05 1.002e-01 100000.0
traitact.AREA_TYPEf:AREAf          0.02179 5.975e-05 8.907e-02  32227.9
traitsoc.AREA_TYPEf:AREAf          0.03635 8.260e-05 1.307e-01  63746.1
traitavo.AREA_TYPEf:AREAf          0.01596 6.780e-05 6.290e-02 100000.0
traitale.AREA_TYPEf:AREAf          0.02946 4.761e-05 1.106e-01  80000.2
traitoth.AREA_TYPEf:AREAf          0.05565 8.772e-05 1.925e-01  46314.4
traitSURVf.2.AREA_TYPEf:AREAf     52.61433 6.910e-05 2.022e+02  24825.3
traitREPRODf.2.AREA_TYPEf:AREAf  225.80887 7.117e-05 1.075e+03    920.4
```

R-structure: ~us(trait):units **(Within-individual variance - covariance estimates)**

```
                                     post.mean l-95% CI u-95% CI eff.samp
traitshy:traitshy.units              0.4616720  0.34475  0.58965    27469
traitexp:traitshy.units             -0.3133409 -0.42297 -0.20610    17882
traitact:traitshy.units             -0.3733016 -0.48859 -0.26419    32675
traitsoc:traitshy.units             -0.0140940 -0.12587  0.09501    18737
traitavo:traitshy.units              0.0712017 -0.03651  0.18351    18525
traitale:traitshy.units             -0.0366033 -0.13867  0.06162    23114
traitoth:traitshy.units             -0.1789147 -0.28266 -0.07837    25427
traitSURVf.2:traitshy.units          0.0125992 -0.30015  0.32600    11068
traitREPRODf.2:traitshy.units        0.0025370 -0.31548  0.32363    11727
traitshy:traitexp.units             -0.3133409 -0.42297 -0.20610    17882
traitexp:traitexp.units              0.9036278  0.74909  1.06151    14871
traitact:traitexp.units              0.1220173  0.02117  0.22373    28232
traitsoc:traitexp.units              0.0047674 -0.10674  0.11712     6957
traitavo:traitexp.units             -0.0549442 -0.16820  0.05754    11626
traitale:traitexp.units             -0.0413917 -0.14730  0.06412    13142
traitoth:traitexp.units              0.1554558  0.05050  0.26194    14659
traitSURVf.2:traitexp.units         -0.0243910 -0.47248  0.42116     9565
traitREPRODf.2:traitexp.units        0.0065987 -0.44201  0.44019    10734
traitshy:traitact.units             -0.3733016 -0.48859 -0.26419    32675
traitexp:traitact.units              0.1220173  0.02117  0.22373    28232
traitact:traitact.units              0.4573500  0.33817  0.58295    33819
traitsoc:traitact.units              0.0024477 -0.10737  0.11566    18386
traitavo:traitact.units             -0.0428256 -0.15625  0.06536    17620
traitale:traitact.units              0.0720882 -0.02723  0.17550    28663
traitoth:traitact.units              0.1502169  0.05113  0.25078    24453
traitSURVf.2:traitact.units         -0.0045010 -0.30029  0.30357    13428
traitREPRODf.2:traitact.units       -0.0062929 -0.31214  0.29324    13362
traitshy:traitsoc.units             -0.0140940 -0.12587  0.09501    18737
traitexp:traitsoc.units              0.0047674 -0.10674  0.11712     6957
traitact:traitsoc.units              0.0024477 -0.10737  0.11566    18386
traitsoc:traitsoc.units              0.8081697  0.62446  0.98774    10787
traitavo:traitsoc.units             -0.7515642 -0.91928 -0.58670     9285
traitale:traitsoc.units             -0.0916978 -0.20341  0.01899     8723
traitoth:traitsoc.units              0.0423937 -0.07069  0.15485    17748
traitSURVf.2:traitsoc.units         -0.0091680 -0.43394  0.40926    10060
traitREPRODf.2:traitsoc.units       -0.0003661 -0.42462  0.42577     9804
traitshy:traitavo.units              0.0712017 -0.03651  0.18351    18525
traitexp:traitavo.units             -0.0549442 -0.16820  0.05754    11626
traitact:traitavo.units             -0.0428256 -0.15625  0.06536    17620
traitsoc:traitavo.units             -0.7515642 -0.91928 -0.58670     9285
traitavo:traitavo.units              0.8702089  0.69263  1.05544    10673
traitale:traitavo.units             -0.0768529 -0.18916  0.03315    11299
traitoth:traitavo.units             -0.1803029 -0.29706 -0.06641    18250
traitSURVf.2:traitavo.units          0.0103367 -0.43185  0.44956     9292
traitREPRODf.2:traitavo.units        0.0020867 -0.44322  0.44993     9287
traitshy:traitale.units             -0.0366033 -0.13867  0.06162    23114
traitexp:traitale.units             -0.0413917 -0.14730  0.06412    13142
traitact:traitale.units              0.0720882 -0.02723  0.17550    28663
traitsoc:traitale.units             -0.0916978 -0.20341  0.01899     8723
traitavo:traitale.units             -0.0768529 -0.18916  0.03315    11299
```

traitale:traitale.units	0.8580901	0.70474	1.00935	4465
traitoth:traitale.units	0.0051404	-0.09710	0.10781	11424
traitSURVf.2:traitale.units	0.0065600	-0.42760	0.43629	10587
traitREPRODf.2:traitale.units	0.0016861	-0.43173	0.44670	9893
traitshy:traitoth.units	-0.1789147	-0.28266	-0.07837	25427
traitexp:traitoth.units	0.1554558	0.05050	0.26194	14659
traitact:traitoth.units	0.1502169	0.05113	0.25078	24453
traitsoc:traitoth.units	0.0423937	-0.07069	0.15485	17748
traitavo:traitoth.units	-0.1803029	-0.29706	-0.06641	18250
traitale:traitoth.units	0.0051404	-0.09710	0.10781	11424
traitoth:traitoth.units	0.6073646	0.46953	0.75306	23809
traitSURVf.2:traitoth.units	-0.0051893	-0.36028	0.35458	11946
traitREPRODf.2:traitoth.units	-0.0068093	-0.35933	0.35094	11643
traitshy:traitSURVf.2.units	0.0125992	-0.30015	0.32600	11068
traitexp:traitSURVf.2.units	-0.0243910	-0.47248	0.42116	9565
traitact:traitSURVf.2.units	-0.0045010	-0.30029	0.30357	13428
traitsoc:traitSURVf.2.units	-0.0091680	-0.43394	0.40926	10060
traitavo:traitSURVf.2.units	0.0103367	-0.43185	0.44956	9292
traitale:traitSURVf.2.units	0.0065600	-0.42760	0.43629	10587
traitoth:traitSURVf.2.units	-0.0051893	-0.36028	0.35458	11946
traitSURVf.2:traitSURVf.2.units	0.5000000	0.50000	0.50000	0
traitREPRODf.2:traitSURVf.2.units	0.0000000	0.00000	0.00000	0
traitshy:traitREPRODf.2.units	0.0025370	-0.31548	0.32363	11727
traitexp:traitREPRODf.2.units	0.0065987	-0.44201	0.44019	10734
traitact:traitREPRODf.2.units	-0.0062929	-0.31214	0.29324	13362
traitsoc:traitREPRODf.2.units	-0.0003661	-0.42462	0.42577	9804
traitavo:traitREPRODf.2.units	0.0020867	-0.44322	0.44993	9287
traitale:traitREPRODf.2.units	0.0016861	-0.43173	0.44670	9893
traitoth:traitREPRODf.2.units	-0.0068093	-0.35933	0.35094	11643
traitSURVf.2:traitREPRODf.2.units	0.0000000	0.00000	0.00000	0
traitREPRODf.2:traitREPRODf.2.units	0.5000000	0.50000	0.50000	0

Model output fixed effects

Location effects: cbind(shy, exp, act, soc, avo, ale, oth, SURVf, REPRODf) ~ trait - 1 + trait:AREA_TYPEf + trait:YEARf + trait:bm + at.level(trait, c(1:8)):SEXf + at.level(trait, c(1:7)):ORDERf

	post.mean	l-95% CI	u-95% CI	eff.samp	pMCMC	
traitshy	0.65639	0.12031	1.19557	17936.34	0.01698	*
traitexp	-0.17769	-0.78388	0.40779	27639.41	0.55210	
traitact	-0.93399	-1.47149	-0.40338	19535.26	0.00110	**
traitsoc	-0.06482	-0.65524	0.52333	21722.83	0.82600	
traitavo	-0.10746	-0.69323	0.48221	26213.01	0.71398	
traitale	0.51719	-0.06215	1.10013	27216.56	0.07940	.
traitoth	-0.42562	-1.01836	0.16052	15034.75	0.14770	
traitSURVf.2	62.68016	4.47382	120.46134	10.63	0.00014	***
traitREPRODf.2	54.05420	3.75231	114.48889	11.43	0.00200	**
Effects of area-type on personality traits, survival and reproduction						
traitshy:AREA_TYPEfRED-GREY	0.05769	-0.29188	0.39103	49130.15	0.71186	
traitexp:AREA_TYPEfRED-GREY	-0.09967	-0.47704	0.25838	45777.57	0.56450	
traitact:AREA_TYPEfRED-GREY	-0.08439	-0.45418	0.25663	53865.60	0.59364	
**traitsoc:AREA_TYPEfRED-GREY**	**0.44305**	**0.05891**	**0.83266**	**51963.19**	**0.03356**	*
traitavo:AREA_TYPEfRED-GREY	-0.16507	-0.49277	0.16231	47610.14	0.28744	
traitale:AREA_TYPEfRED-GREY	0.13000	-0.24675	0.48678	47223.17	0.42016	
traitoth:AREA_TYPEfRED-GREY	-0.24142	-0.69213	0.22771	64670.87	0.24118	
traitSURVf.2:AREA_TYPEfRED-GREY	-14.84952	-53.21532	19.05063	319.54	0.31390	
traitREPRODf.2:AREA_TYPEfRED-GREY	-27.88988	-83.21459	17.76288	34.43	0.15696	
Effect of year on personality traits, survival and reproduction						
traitshy:YEARf2017	-0.05552	-0.29558	0.18809	8877.22	0.65190	
traitexp:YEARf2017	-0.30783	-0.55410	-0.05641	12280.16	0.01578	*
traitact:YEARf2017	0.31719	0.08163	0.55549	11513.86	0.00910	**
traitsoc:YEARf2017	0.20547	-0.03238	0.44985	14096.84	0.09448	.

```
traitavo:YEARf2017                 -0.13407  -0.38570   0.11727 17301.36 0.29360
traitale:YEARf2017                 -0.80213  -1.04803  -0.55562  7233.28 < 1e-05 ***
traitoth:YEARf2017                  0.79276   0.54565   1.03126  7418.18 < 1e-05 ***
traitSURVf.2:YEARf2017            -41.57733 -83.38803  -2.05363    10.53 < 1e-05 ***
traitREPRODf.2:YEARf2017          -48.10580 -91.29551 -20.27583    12.73 < 1e-05 ***

Effect of body mass on personality traits, survival and reproduction
traitshy:bm                         0.02478  -0.10196   0.15177  6669.84 0.69900
traitexp:bm                         0.06359  -0.06601   0.19064 10636.59 0.33136
traitact:bm                        -0.05100  -0.17626   0.07189  8357.44 0.41626
traitsoc:bm                         0.08286  -0.04256   0.21067  8494.08 0.19984
traitavo:bm                        -0.09303  -0.22360   0.03810 11781.48 0.16466
traitale:bm                        -0.10163  -0.22667   0.02340 10890.50 0.11282
traitoth:bm                         0.03927  -0.08714   0.16389  7454.52 0.53778
traitSURVf.2:bm                     4.89446  -9.46027  19.42876    72.14 0.37504
traitREPRODf.2:bm                  43.51851  15.09151  81.92606    10.68 < 1e-05 ***

Effects of sex on personality traits, survival
at.level(shy,   c(1:8))1:SEXfF     -0.15368  -0.43144   0.13324  5534.62 0.28534
at.level(exp,   c(1:8))2:SEXfF      0.01153  -0.24372   0.27297  7520.94 0.92724
at.level(act,   c(1:8))3:SEXfF      0.19262  -0.07366   0.46744 11603.36 0.16204
at.level(soc,   c(1:8))4:SEXfF      0.22719  -0.02409   0.47925  7830.94 0.07714 .
at.level(avo,   c(1:8))5:SEXfF     -0.28581  -0.54798  -0.02406 10430.18 0.03342 *
at.level(ale,   c(1:8))6:SEXfF      0.10449  -0.14642   0.34668 19373.17 0.40356
at.level(oth,   c(1:8))7:SEXfF     -0.01717  -0.28002   0.24379 12954.82 0.89804
at.level(surv,  c(1:8))8:SEXfF     15.84768 -15.54971  57.17736   117.49 0.31248

Effects of arena test order on personality traits
at.level(shy,   c(1:7))1:ORDERf1   -0.87544  -1.32666  -0.42677 20777.73   4e-05 ***
at.level(exp,   c(1:7))2:ORDERf1    0.54066   0.01042   1.07478 29438.12 0.04606 *
at.level(act,   c(1:7))3:ORDERf1    1.06456   0.62742   1.51126 24252.84 < 1e-05 ***
at.level(soc,   c(1:7))4:ORDERf1   -0.41545  -0.92470   0.10950 19312.72 0.11414
at.level(avo,   c(1:7))5:ORDERf1    0.39240  -0.13828   0.93162 25535.35 0.14952
at.level(ale,   c(1:7))6:ORDERf1   -0.26670  -0.79533   0.25055 27002.28 0.31580
at.level(oth,   c(1:7))7:ORDERf1    0.26859  -0.20123   0.74917 14967.20 0.26858
at.level(shy,   c(1:7))1:ORDERf2   -0.25738  -0.69076   0.17711 28459.12 0.24252
at.level(exp,   c(1:7))2:ORDERf2    0.26399  -0.27441   0.80047 37344.04 0.33714
at.level(act,   c(1:7))3:ORDERf2    0.38067  -0.04054   0.81631 32930.05 0.08256 .
at.level(soc,   c(1:7))4:ORDERf2   -0.34727  -0.86963   0.16651 28827.45 0.18680
at.level(avo,   c(1:7))5:ORDERf2    0.40793  -0.11313   0.95534 37698.42 0.13378
at.level(ale,   c(1:7))6:ORDERf2   -0.24636  -0.77596   0.27790 29987.98 0.35564
at.level(oth,   c(1:7))7:ORDERf2    0.10341  -0.36893   0.57032 21252.08 0.66766
at.level(shy,   c(1:7))1:ORDERf3   -0.30437  -0.74159   0.11671 72607.33 0.16062
at.level(exp,   c(1:7))2:ORDERf3    0.18334  -0.40073   0.75472 74906.46 0.53578
at.level(act,   c(1:7))3:ORDERf3    0.39945  -0.03291   0.81591 76331.05 0.06446 .
at.level(soc,   c(1:7))4:ORDERf3   -0.35490  -0.91516   0.18856 47462.80 0.20588
at.level(avo,   c(1:7))5:ORDERf3    0.36250  -0.20339   0.94140 70747.32 0.21194
at.level(ale,   c(1:7))6:ORDERf3    0.01100  -0.55600   0.57640 54584.64 0.96976
at.level(oth,   c(1:7))7:ORDERf3    0.02251  -0.45530   0.50750 52131.55 0.93062
```

Correlation analysis

correlation with survival

```
Correlation estimates for shyness-survival
        Mean: 0.03274341
        Marginal parameters posterior mode: 0.01742753
        Highest Posterior Density interval: -0.21429 0.2823377

Correlation estimates for exploration-survival
        Mean: -0.2214111
        Marginal parameters posterior mode: -0.2525842
        Highest Posterior Density interval: -0.7244422 0.3052665
```

```
Correlation estimates for activity-survival
        Mean: -0.02604208
        Marginal parameters posterior mode: -0.02780341
        Highest Posterior Density interval: -0.2821705 0.2309646

Correlation estimates for sociability-survival
        Mean: -0.2450685
        Marginal parameters posterior mode: -0.224419
        Highest Posterior Density interval: -0.7851539 0.2667296

Correlation estimates for avoidance-survival
        Mean: 0.1349096
        Marginal parameters posterior mode: 0.15363
        Highest Posterior Density interval: -0.349693 0.6418636

Correlation estimates for alert-survival
        Mean: 0.2435647
        Marginal parameters posterior mode: 0.3439264
        Highest Posterior Density interval: -0.3962007 0.8310232

Correlation estimates for other-survival
        Mean: -0.1321872
        Marginal parameters posterior mode: -0.1532009
        Highest Posterior Density interval: -0.4552511 0.2010649
```

correlation with reproduction

```
Correlation estimates for shyness-reproduction
        Mean: -0.3357251
        Marginal parameters posterior mode: -0.312562
        Highest Posterior Density interval: -0.7351616 0.02010848

Correlation estimates for exploration-reproduction
        Mean: 0.4722743
        Marginal parameters posterior mode: 0.6104905
        Highest Posterior Density interval: -0.06938685 0.9342063

Correlation estimates for activity-reproduction
        Mean: 0.2878644
        Marginal parameters posterior mode: 0.1954553
        Highest Posterior Density interval: -0.1056873 0.7654844

Correlation estimates for sociability-reproduction
        Mean: 0.2445107
        Marginal parameters posterior mode: 0.495825
        Highest Posterior Density interval: -0.6334388 0.9180481

Correlation estimates for avoidance-reproduction
        Mean: -0.3154024
        Marginal parameters posterior mode: -0.41096
        Highest Posterior Density interval: -0.9093162 0.372889


Correlation estimates for alert-reproduction
        Mean: 0.4662205
        Marginal parameters posterior mode: 0.8624024
        Highest Posterior Density interval: -0.2393639 0.983493

Correlation estimates for other-reproduction
        Mean: 0.3330506
        Marginal parameters posterior mode: 0.2472986
        Highest Posterior Density interval: -0.1138344 0.7880943
```

Note: The personality trait avoidance was expressed slightly more by males than by females. In both coniferous or deciduous habitats, red squirrels display intra-sexual territoriality among adult females, while male space use is characterized by both intra- and intersexual core-area overlap based on a dominance hierarchy related to age and body mass (Di Pierro, Molinari, Tosi, & Wauters, 2008; Wauters & Dhondt, 1992). Amicable social behaviours are rare in the field and animals foraging in close proximity tend to avoid or chase each other (e.g. Wauters & Dhondt, 1987; Wauters & Gurnell, 1999). For males the decision to chase or avoid interactions might depend more strongly from cues related to the potential opponent than for the more territorial females, which could explain why in MIS males tend to avoid more their mirror image than females.

Table S4. Correlation matrix (r values and 95% Credibility Intervals based on the MCMCglmm model in Table S3) between the personality trait scores of red squirrels. Significant correlations in bold.

Trait	Exploration	Shyness	Sociability	Avoidance	Other	Alert
Activity	**0.68 (0.31 – 0.97)**	**-0.97 (-0.99 - -0.93)**	0.26 (-0.27 – 0.81)	**-0.60 (-0.96 - -0.20)**	**0.91 (0.80 – 0.99)**	0.47 (-0.03 – 0.94)
Exploration		**-0.73 (-0.98 - -0.40)**	0.40 (-0.23 – 0.93)	**-0.58 (-0.97 – -0.07)**	**0.72 (0.37 – 0.98)**	0.34 (-0.27 – 0.91)
Shyness			-0.28 (-0.82 – 0.24)	**0.65 (0.29 – 0.97)**	**-0.91 (-0.99 - -0.81)**	-0.42 (-0.91 – 0.11)
Sociability				**-0.83 (-0.98 - -0.59)**	0.28 (-0.26 – 0.86)	0.04 (-0.65 – 0.77)
Avoidance					**-0.63 (-0.97 - -0.23)**	-0.24 (-0.89 – 0.42)
Other						0.45 (-0.09 – 0.93)

Activity and shyness are strongly negatively correlated indicating they are the two extremes of an activity-shyness axis. Activity is positively correlated with exploration suggesting a behavioral syndrome of active squirrels having also stronger tendency to explore than less active ones (see also Santicchia et al., 2018a). MIS revealed a negative correlation between sociability and avoidance. Squirrels expressing high activity and exploration in OFT tended to show low avoidance in MIS, while animals defined as shy during OFT showed high avoidance during MIS.

Table S5. MCMCglmm outputs of the two submodels, one for Red-grey sites and one for the Red-only sites calculated to allow estimates of interactions of the dependent variables with area-type (for detailed description see Statistical Analysis).

1) **Red-Grey sites summary**

```
----------------------
Iterations = 50001:1049961
Thinning interval  = 40
Sample size  = 25000

DIC: 1499.768

G-structure:  ~us(trait):IDf

                                   post.mean   l-95% CI   u-95% CI eff.samp
traitshy:traitshy.IDf                0.45223   0.208166  7.112e-01 6113.549
traitexp:traitshy.IDf               -0.14384  -0.307048  6.561e-03 3065.567
traitact:traitshy.IDf               -0.37287  -0.602489 -1.585e-01 5723.317
traitsoc:traitshy.IDf               -0.10075  -0.299890  1.051e-01 3572.432
traitavo:traitshy.IDf                0.19824  -0.002452  3.960e-01 4176.013
traitale:traitshy.IDf               -0.06017  -0.221851  9.015e-02 2857.684
traitoth:traitshy.IDf               -0.30312  -0.499942 -1.164e-01 5566.443
traitSURVf.2:traitshy.IDf            0.86604  -5.923667  7.986e+00  431.120
traitREPRODf.2:traitshy.IDf          0.68930  -3.888469  6.304e+00   37.643
traitshy:traitexp.IDf               -0.14384  -0.307048  6.561e-03 3065.567
traitexp:traitexp.IDf                0.10709   0.010515  2.264e-01 1977.117
traitact:traitexp.IDf                0.11341  -0.011554  2.565e-01 2877.584
traitsoc:traitexp.IDf                0.04812  -0.111978  2.039e-01 1507.582
traitavo:traitexp.IDf               -0.07292  -0.224460  8.522e-02 1606.403
traitale:traitexp.IDf                0.01050  -0.080630  9.974e-02 2033.799
traitoth:traitexp.IDf                0.10867  -0.015285  2.448e-01 3631.313
traitSURVf.2:traitexp.IDf           -1.52140  -8.436602  5.364e+00  305.958
traitREPRODf.2:traitexp.IDf         -1.01577  -5.205388  2.890e+00  121.466
traitshy:traitact.IDf               -0.37287  -0.602489 -1.585e-01 5723.317
traitexp:traitact.IDf                0.11341  -0.011554  2.565e-01 2877.584
traitact:traitact.IDf                0.33188   0.134563  5.562e-01 5346.001
traitsoc:traitact.IDf                0.05260  -0.129401  2.424e-01 2817.877
traitavo:traitact.IDf               -0.13031  -0.313514  5.197e-02 3279.958
traitale:traitact.IDf                0.06436  -0.068958  2.117e-01 1987.136
traitoth:traitact.IDf                0.24830   0.084020  4.242e-01 5422.063
traitSURVf.2:traitact.IDf           -1.86742  -9.144072  4.243e+00  217.058
traitREPRODf.2:traitact.IDf         -0.80456  -5.668689  3.342e+00   44.501
traitshy:traitsoc.IDf               -0.10075  -0.299890  1.051e-01 3572.432
traitexp:traitsoc.IDf                0.04812  -0.111978  2.039e-01 1507.582
traitact:traitsoc.IDf                0.05260  -0.129401  2.424e-01 2817.877
traitsoc:traitsoc.IDf                0.41585   0.105552  7.357e-01 1680.673
traitavo:traitsoc.IDf               -0.39987  -0.700401 -1.169e-01 1927.052
traitale:traitsoc.IDf               -0.06594  -0.238523  9.222e-02 1218.325
traitoth:traitsoc.IDf                0.06748  -0.124931  2.567e-01 1860.769
traitSURVf.2:traitsoc.IDf           -1.26677 -10.257139  6.306e+00  155.973
traitREPRODf.2:traitsoc.IDf         -2.60096  -7.804144  2.134e+00   60.441
traitshy:traitavo.IDf                0.19824  -0.002452  3.960e-01 4176.013
traitexp:traitavo.IDf               -0.07292  -0.224460  8.522e-02 1606.403
traitact:traitavo.IDf               -0.13031  -0.313514  5.197e-02 3279.958
traitsoc:traitavo.IDf               -0.39987  -0.700401 -1.169e-01 1927.052
traitavo:traitavo.IDf                0.43101   0.159166  7.410e-01 2328.935
traitale:traitavo.IDf                0.03702  -0.123702  1.930e-01 1385.083
traitoth:traitavo.IDf               -0.14236  -0.328944  4.513e-02 2211.300
traitSURVf.2:traitavo.IDf            0.86244  -6.934658  9.415e+00  204.778
traitREPRODf.2:traitavo.IDf          2.38875  -2.729009  7.659e+00   56.429
traitshy:traitale.IDf               -0.06017  -0.221851  9.015e-02 2857.684
traitexp:traitale.IDf                0.01050  -0.080630  9.974e-02 2033.799
traitact:traitale.IDf                0.06436  -0.068958  2.117e-01 1987.136
traitsoc:traitale.IDf               -0.06594  -0.238523  9.222e-02 1218.325
traitavo:traitale.IDf                0.03702  -0.123702  1.930e-01 1385.083
traitale:traitale.IDf                0.10363   0.006913  2.320e-01 1350.006
traitoth:traitale.IDf                0.03757  -0.091681  1.690e-01 2651.056
traitSURVf.2:traitale.IDf           -0.73034  -8.955434  6.429e+00  235.501
traitREPRODf.2:traitale.IDf          0.30015  -3.648798  4.111e+00  226.565
traitshy:traitoth.IDf               -0.30312  -0.499942 -1.164e-01 5566.443
traitexp:traitoth.IDf                0.10867  -0.015285  2.448e-01 3631.313
```

```
traitact:traitoth.IDf              0.24830   0.084020  4.242e-01 5422.063
traitsoc:traitoth.IDf              0.06748  -0.124931  2.567e-01 1860.769
traitavo:traitoth.IDf             -0.14236  -0.328944  4.513e-02 2211.300
traitale:traitoth.IDf              0.03757  -0.091681  1.690e-01 2651.056
traitoth:traitoth.IDf              0.29826   0.104176  5.170e-01 3177.282
traitSURVf.2:traitoth.IDf         -3.16725 -11.302993  3.224e+00   85.608
traitREPRODf.2:traitoth.IDf       -1.33080  -5.943188  2.875e+00   46.007
traitshy:traitSURVf.2.IDf          0.86604  -5.923667  7.986e+00  431.120
traitexp:traitSURVf.2.IDf         -1.52140  -8.436602  5.364e+00  305.958
traitact:traitSURVf.2.IDf         -1.86742  -9.144072  4.243e+00  217.058
traitsoc:traitSURVf.2.IDf         -1.26677 -10.257139  6.306e+00  155.973
traitavo:traitSURVf.2.IDf          0.86244  -6.934658  9.415e+00  204.778
traitale:traitSURVf.2.IDf         -0.73034  -8.955434  6.429e+00  235.501
traitoth:traitSURVf.2.IDf         -3.16725 -11.302993  3.224e+00   85.608
traitSURVf.2:traitSURVf.2.IDf    859.43446  15.254605  1.770e+03    3.565
traitREPRODf.2:traitSURVf.2.IDf  351.78860   2.781029  7.299e+02    4.787
traitshy:traitREPRODf.2.IDf        0.68930  -3.888469  6.304e+00   37.643
traitexp:traitREPRODf.2.IDf       -1.01577  -5.205388  2.890e+00  121.466
traitact:traitREPRODf.2.IDf       -0.80456  -5.668689  3.342e+00   44.501
traitsoc:traitREPRODf.2.IDf       -2.60096  -7.804144  2.134e+00   60.441
traitavo:traitREPRODf.2.IDf        2.38875  -2.729009  7.659e+00   56.429
traitale:traitREPRODf.2.IDf        0.30015  -3.648798  4.111e+00  226.565
traitoth:traitREPRODf.2.IDf       -1.33080  -5.943188  2.875e+00   46.007
traitSURVf.2:traitREPRODf.2.IDf  351.78860   2.781029  7.299e+02    4.787
traitREPRODf.2:traitREPRODf.2.IDf 222.76316   2.078551  4.646e+02    7.113

               ~idh(trait):AREAf

                     post.mean  l-95% CI u-95% CI eff.samp
traitshy.AREAf          0.9742 8.521e-05   1.7267    25000
traitexp.AREAf          0.2728 5.936e-05   0.4897    21666
traitact.AREAf          2.3359 1.367e-04   3.0049    25000
traitsoc.AREAf          0.2723 7.248e-05   0.5508    25000
traitavo.AREAf          0.1629 6.217e-05   0.2486    25000
traitale.AREAf          0.1705 7.213e-05   0.2719    25000
traitoth.AREAf          1.2385 1.345e-04   2.3548    25000
traitSURVf.2.AREAf     27.3485 8.379e-05  47.4123    24393
traitREPRODf.2.AREAf   15.9995 6.534e-05  24.0382    25000

 R-structure:  ~us(trait):units

                                    post.mean  l-95% CI u-95% CI eff.samp
traitshy:traitshy.units              0.447889  0.294803  0.61708     7716
traitexp:traitshy.units             -0.240988 -0.384656 -0.10325     4658
traitact:traitshy.units             -0.371334 -0.536607 -0.23203     7100
traitsoc:traitshy.units              0.052153 -0.101461  0.20397     4152
traitavo:traitshy.units             -0.035520 -0.182183  0.10845     3838
traitale:traitshy.units             -0.080340 -0.228210  0.06199     4361
traitoth:traitshy.units             -0.127886 -0.260058  0.00193     6732
traitSURVf.2:traitshy.units          0.019308 -0.301735  0.34059     2143
traitREPRODf.2:traitshy.units       -0.021091 -0.345012  0.31020     2062
traitshy:traitexp.units             -0.240988 -0.384656 -0.10325     4658
traitexp:traitexp.units              0.833737  0.629219  1.04480     9231
traitact:traitexp.units              0.061862 -0.071393  0.19846     4529
traitsoc:traitexp.units             -0.030817 -0.193302  0.12770     2843
traitavo:traitexp.units             -0.003135 -0.164709  0.15122     2986
traitale:traitexp.units             -0.038338 -0.196261  0.11369    10237
traitoth:traitexp.units              0.141438  0.005426  0.28817     6443
traitSURVf.2:traitexp.units         -0.053996 -0.484989  0.39218     1674
traitREPRODf.2:traitexp.units        0.017605 -0.420790  0.46365     1631
traitshy:traitact.units             -0.371334 -0.536607 -0.23203     7100
traitexp:traitact.units              0.061862 -0.071393  0.19846     4529
traitact:traitact.units              0.474945  0.316985  0.65273     5837
traitsoc:traitact.units             -0.018083 -0.170347  0.13805     4000
traitavo:traitact.units              0.007561 -0.141849  0.15406     3827
traitale:traitact.units              0.095999 -0.044548  0.25028     3652
traitoth:traitact.units              0.123915 -0.004036  0.25349     7079
traitSURVf.2:traitact.units         -0.007376 -0.333857  0.31971     2528
traitREPRODf.2:traitact.units        0.020340 -0.309527  0.33909     2209
traitshy:traitsoc.units              0.052153 -0.101461  0.20397     4152
traitexp:traitsoc.units             -0.030817 -0.193302  0.12770     2843
traitact:traitsoc.units             -0.018083 -0.170347  0.13805     4000
traitsoc:traitsoc.units              0.686949  0.425366  0.96520     2087
```

```
traitavo:traitsoc.units             -0.598392 -0.856051 -0.38534     2160
traitale:traitsoc.units             -0.072898 -0.249699  0.10093     2186
traitoth:traitsoc.units             -0.024109 -0.177664  0.13500     2642
traitSURVf.2:traitsoc.units         -0.034597 -0.442065  0.39897     1791
traitREPRODf.2:traitsoc.units        0.030528 -0.385554  0.44631     1668
traitshy:traitavo.units             -0.035520 -0.182183  0.10845     3838
traitexp:traitavo.units             -0.003135 -0.164709  0.15122     2986
traitact:traitavo.units              0.007561 -0.141849  0.15406     3827
traitsoc:traitavo.units             -0.598392 -0.856051 -0.38534     2160
traitavo:traitavo.units              0.720863  0.485039  0.97843     2939
traitale:traitavo.units             -0.138493 -0.310527  0.03034     2524
traitoth:traitavo.units             -0.071196 -0.221455  0.07887     3463
traitSURVf.2:traitavo.units          0.036178 -0.401338  0.45641     1702
traitREPRODf.2:traitavo.units       -0.043102 -0.459790  0.38670     1574
traitshy:traitale.units             -0.080340 -0.228210  0.06199     4361
traitexp:traitale.units             -0.038338 -0.196261  0.11369    10237
traitact:traitale.units              0.095999 -0.044548  0.25028     3652
traitsoc:traitale.units             -0.072898 -0.249699  0.10093     2186
traitavo:traitale.units             -0.138493 -0.310527  0.03034     2524
traitale:traitale.units              0.930873  0.717915  1.18107     5483
traitoth:traitale.units              0.032810 -0.111163  0.17672     6171
traitSURVf.2:traitale.units         -0.003959 -0.477137  0.48121     1746
traitREPRODf.2:traitale.units        0.039702 -0.445791  0.49784     1451
traitshy:traitoth.units             -0.127886 -0.260058  0.00193     6732
traitexp:traitoth.units              0.141438  0.005426  0.28817     6443
traitact:traitoth.units              0.123915 -0.004036  0.25349     7079
traitsoc:traitoth.units             -0.024109 -0.177664  0.13500     2642
traitavo:traitoth.units             -0.071196 -0.221455  0.07887     3463
traitale:traitoth.units              0.032810 -0.111163  0.17672     6171
traitoth:traitoth.units              0.537011  0.366568  0.72542     4256
traitSURVf.2:traitoth.units         -0.009837 -0.368810  0.33970     2319
traitREPRODf.2:traitoth.units        0.039557 -0.324033  0.37352     1838
traitshy:traitSURVf.2.units          0.019308 -0.301735  0.34059     2143
traitexp:traitSURVf.2.units         -0.053996 -0.484989  0.39218     1674
traitact:traitSURVf.2.units         -0.007376 -0.333857  0.31971     2528
traitsoc:traitSURVf.2.units         -0.034597 -0.442065  0.39897     1791
traitavo:traitSURVf.2.units          0.036178 -0.401338  0.45641     1702
traitale:traitSURVf.2.units         -0.003959 -0.477137  0.48121     1746
traitoth:traitSURVf.2.units         -0.009837 -0.368810  0.33970     2319
traitSURVf.2:traitSURVf.2.units      0.500000  0.500000  0.50000        0
traitREPRODf.2:traitSURVf.2.units    0.000000  0.000000  0.00000        0
traitshy:traitREPRODf.2.units       -0.021091 -0.345012  0.31020     2062
traitexp:traitREPRODf.2.units        0.017605 -0.420790  0.46365     1631
traitact:traitREPRODf.2.units        0.020340 -0.309527  0.33909     2209
traitsoc:traitREPRODf.2.units        0.030528 -0.385554  0.44631     1668
traitavo:traitREPRODf.2.units       -0.043102 -0.459790  0.38670     1574
traitale:traitREPRODf.2.units        0.039702 -0.445791  0.49784     1451
traitoth:traitREPRODf.2.units        0.039557 -0.324033  0.37352     1838
traitSURVf.2:traitREPRODf.2.units    0.000000  0.000000  0.00000        0
traitREPRODf.2:traitREPRODf.2.units  0.500000  0.500000  0.50000        0

Location effects: cbind(shy, exp, act, soc, avo, ale, oth, SURVf, REPRODf)
~ trait - 1 + trait:YEARf + trait:bm + at.level(trait, c(1:8)):SEXf +
at.level(trait, c(1:7)):ORDERf

                                  post.mean   l-95% CI   u-95% CI eff.samp   pMCMC
traitshy                           0.346737  -0.623397   1.362666 10537.38 0.43816
traitexp                           0.574042  -0.303384   1.455607  6933.84 0.18424
traitact                          -1.004893  -2.124848   0.160790 23852.94 0.06776 .
traitsoc                          -0.152718  -1.067087   0.767008  4078.06 0.73008
traitavo                           0.100787  -0.770562   0.982556  3792.38 0.81328
traitale                           0.405666  -0.463115   1.263920 10773.48 0.35192
traitoth                          -0.121668  -1.205073   0.929594 13690.52 0.80520
traitSURVf.2                      15.619601   1.570770  29.618376    17.65 0.00840 **
traitREPRODf.2                     9.292958   0.876821  17.702782    24.62 0.01392 *
traitshy:YEARf2017                 0.121557  -0.244491   0.467962  1730.52 0.50368
traitexp:YEARf2017                -1.108426  -1.461601  -0.760858  6845.81 < 4e-05 ***
traitact:YEARf2017                 0.503757   0.165455   0.847537  1818.88 0.00520 **
traitsoc:YEARf2017                 0.184787  -0.193505   0.551690  1535.49 0.33072
traitavo:YEARf2017                -0.066917  -0.435642   0.313044  1527.36 0.72104
traitale:YEARf2017                -0.538437  -0.907734  -0.190492  6168.33 0.00424 **
traitoth:YEARf2017                 0.692412   0.351986   1.032533  2800.58 < 4e-05 ***
traitSURVf.2:YEARf2017           -13.473165 -24.019416  -1.126401    15.70 0.00088 ***
```

```
traitREPRODf.2:YEARf2017        -15.095600 -26.302849  -3.548984    11.92 0.00056 ***
traitshy:bm                       0.066327  -0.118193   0.235195  5557.90 0.46112
traitexp:bm                       0.003173  -0.173628   0.183141  9472.29 0.96664
traitact:bm                      -0.026641  -0.191822   0.149788  5777.09 0.76160
traitsoc:bm                       0.115250  -0.079092   0.314374  3218.33 0.26032
traitavo:bm                      -0.119380  -0.315427   0.082363  3912.81 0.23736
traitale:bm                      -0.151250  -0.339749   0.037604 13102.77 0.11216
traitoth:bm                       0.010104  -0.159341   0.182861  5486.92 0.90920
traitSURVf.2:bm                   0.197193  -6.818316   7.446056    36.57 0.92360
traitREPRODf.2:bm                11.903997   3.397128  18.845164    14.60 < 4e-05 ***
at.level(trait, c(1:8))1:SEXfF   -0.233876  -0.586801   0.131086  5436.31 0.20168
at.level(trait, c(1:8))2:SEXfF    0.101325  -0.223025   0.431025  7671.72 0.53784
at.level(trait, c(1:8))3:SEXfF    0.163528  -0.169043   0.503771  5059.85 0.33208
at.level(trait, c(1:8))4:SEXfF    0.441338   0.052591   0.829225  3502.01 0.02624 *
at.level(trait, c(1:8))5:SEXfF   -0.489774  -0.880947  -0.091996  3660.74 0.01560 *
at.level(trait, c(1:8))6:SEXfF    0.160428  -0.184485   0.494966 19406.24 0.35440
at.level(trait, c(1:8))7:SEXfF    0.035042  -0.290760   0.379344  7201.79 0.83312
at.level(trait, c(1:8))8:SEXfF    1.297157 -10.222725  14.227834    69.45 0.82088
at.level(trait, c(1:7))1:ORDERf1 -0.605576  -1.235622   0.040786  5067.78 0.06248 .
at.level(trait, c(1:7))2:ORDERf1 -0.037040  -0.812554   0.710633  4798.85 0.92800
at.level(trait, c(1:7))3:ORDERf1  1.069601   0.422443   1.695958  5862.15 0.00056 ***
at.level(trait, c(1:7))4:ORDERf1  0.123340  -0.625891   0.886699  3321.08 0.74624
at.level(trait, c(1:7))5:ORDERf1 -0.017095  -0.781526   0.745803  3403.37 0.96512
at.level(trait, c(1:7))6:ORDERf1 -0.133293  -0.931693   0.632124  8497.02 0.74416
at.level(trait, c(1:7))7:ORDERf1 -0.229615  -0.900973   0.430848  4803.88 0.50152
at.level(trait, c(1:7))1:ORDERf2 -0.087528  -0.703917   0.516738  6260.46 0.77776
at.level(trait, c(1:7))2:ORDERf2 -0.062594  -0.807038   0.685480  7484.21 0.87144
at.level(trait, c(1:7))3:ORDERf2  0.409888  -0.197294   1.019065  7257.06 0.18664
at.level(trait, c(1:7))4:ORDERf2 -0.040151  -0.760730   0.697518  4047.89 0.91208
at.level(trait, c(1:7))5:ORDERf2  0.203927  -0.540920   0.939868  4087.94 0.58424
at.level(trait, c(1:7))6:ORDERf2 -0.107427  -0.885603   0.662220 12261.36 0.78704
at.level(trait, c(1:7))7:ORDERf2 -0.250367  -0.875992   0.401256  7328.51 0.43904
at.level(trait, c(1:7))1:ORDERf3 -0.022180  -0.594297   0.577718 14566.01 0.94240
at.level(trait, c(1:7))2:ORDERf3 -0.256356  -1.020652   0.541961 15096.39 0.52320
at.level(trait, c(1:7))3:ORDERf3  0.107910  -0.486900   0.708794 17363.56 0.71952
at.level(trait, c(1:7))4:ORDERf3 -0.072124  -0.797569   0.655146  8258.43 0.84208
at.level(trait, c(1:7))5:ORDERf3  0.156826  -0.599808   0.886188 10163.26 0.67152
at.level(trait, c(1:7))6:ORDERf3  0.116265  -0.713454   0.913952 15858.71 0.77976
at.level(trait, c(1:7))7:ORDERf3 -0.548719  -1.158044   0.105964 14159.91 0.09416 .
```

Correlation analysis

correlation with survival

```
Correlation estimates for shyness-survival
      Mean: 0.04871845
      Marginal parameters posterior mode: 0.08606807
      Highest Posterior Density interval: -0.282412 0.3881313

Correlation estimates for exploration-survival
      Mean: -0.1932554
      Marginal parameters posterior mode: -0.3180277
      Highest Posterior Density interval: -0.8203025 0.4839247

Correlation estimates for activity-survival
      Mean: -0.1191214
      Marginal parameters posterior mode: -0.09732655
      Highest Posterior Density interval: -0.4944661 0.2441474

Correlation estimates for sociability-survival
      Mean: -0.06168476
      Marginal parameters posterior mode: -0.003457743
      Highest Posterior Density interval: -0.4806388 0.3565331

Correlation estimates for avoidance-survival
      Mean: 0.03805248
      Marginal parameters posterior mode: 0.0006237439
      Highest Posterior Density interval: -0.3767508 0.4259202

Correlation estimates for alert-survival
```

```
      Mean: -0.0879861
      Marginal parameters posterior mode: -0.05722858
      Highest Posterior Density interval: -0.8434349 0.6343751

Correlation estimates for other-survival
      Mean: -0.2093157
      Marginal parameters posterior mode: -0.2560751
      Highest Posterior Density interval: -0.6122165 0.2230251
```

correlation with reproduction

```
Correlation estimates for shyness-reproduction
      Mean: 0.06526915
      Marginal parameters posterior mode: 0.006928147
      Highest Posterior Density interval: -0.4018751 0.5720033

Correlation estimates for exploration-reproduction
      Mean: -0.233665
      Marginal parameters posterior mode: -0.4370985
      Highest Posterior Density interval: -0.9203873 0.476229

Correlation estimates for activity-reproduction
      Mean: -0.08938369
      Marginal parameters posterior mode: 0.006218858
      Highest Posterior Density interval: -0.626288 0.4060403

Correlation estimates for sociability-reproduction
      Mean: -0.2964138
      Marginal parameters posterior mode: -0.3517965
      Highest Posterior Density interval: -0.767487 0.2324247

Correlation estimates for avoidance-reproduction
      Mean: 0.2614671
      Marginal parameters posterior mode: 0.3286697
      Highest Posterior Density interval: -0.278389 0.7261405

Correlation estimates for alert-reproduction
      Mean: 0.07561613
      Marginal parameters posterior mode: 0.1612611
      Highest Posterior Density interval: -0.7131155 0.8202925

Correlation estimates for other-reproduction
      Mean: -0.1687436
      Marginal parameters posterior mode: -0.2102566
      Highest Posterior Density interval: -0.6901834 0.376124
```

2. Red only areas summary

```
----------------------
Iterations = 50001:1049961
Thinning interval  = 40
Sample size  = 25000

DIC: 1527.789

 G-structure:  ~us(trait):IDf

                                post.mean  l-95% CI   u-95% CI eff.samp
traitshy:traitshy.IDf            5.297e-01  2.117e-01  8.847e-01 3943.617
traitexp:traitshy.IDf           -2.708e-01 -4.977e-01 -4.572e-02 1719.021
traitact:traitshy.IDf           -4.799e-01 -7.909e-01 -1.833e-01 4372.810
traitsoc:traitshy.IDf           -5.860e-02 -2.107e-01  8.704e-02 2119.751
traitavo:traitshy.IDf            1.408e-01 -2.867e-02  3.265e-01 1576.750
traitale:traitshy.IDf           -7.758e-02 -2.636e-01  1.055e-01 1014.462
traitoth:traitshy.IDf           -4.094e-01 -6.953e-01 -1.378e-01 2581.700
traitSURVf.2:traitshy.IDf        3.557e+00 -3.317e+00  1.178e+01  124.945
traitREPRODf.2:traitshy.IDf     -1.479e+01 -3.523e+01  2.457e+00   11.033
traitshy:traitexp.IDf           -2.708e-01 -4.977e-01 -4.572e-02 1719.021
```

```
traitexp:traitexp.IDf              2.377e-01  4.962e-02  4.511e-01 1465.441
traitact:traitexp.IDf              2.224e-01  2.651e-02  4.294e-01 1998.522
traitsoc:traitexp.IDf              4.852e-02 -5.037e-02  1.578e-01 1410.132
traitavo:traitexp.IDf             -7.738e-02 -2.064e-01  3.714e-02 1585.550
traitale:traitexp.IDf              3.528e-02 -9.630e-02  1.665e-01 1080.735
traitoth:traitexp.IDf              2.214e-01  2.541e-02  4.260e-01 3085.816
traitSURVf.2:traitexp.IDf         -5.196e+00 -1.499e+01  1.553e+00   39.049
traitREPRODf.2:traitexp.IDf        1.062e+01 -1.025e+00  2.238e+01   49.830
traitshy:traitact.IDf             -4.799e-01 -7.909e-01 -1.833e-01 4372.810
traitexp:traitact.IDf              2.224e-01  2.651e-02  4.294e-01 1998.522
traitact:traitact.IDf              4.911e-01  2.082e-01  7.997e-01 5037.580
traitsoc:traitact.IDf              5.360e-02 -8.365e-02  1.989e-01 2153.132
traitavo:traitact.IDf             -1.360e-01 -3.043e-01  2.763e-02 2203.481
traitale:traitact.IDf              9.802e-02 -6.984e-02  2.776e-01 1574.836
traitoth:traitact.IDf              4.051e-01  1.585e-01  6.793e-01 2619.872
traitSURVf.2:traitact.IDf         -1.531e+00 -8.980e+00  5.264e+00  236.261
traitREPRODf.2:traitact.IDf        1.256e+01 -4.557e+00  3.235e+01   10.031
traitshy:traitsoc.IDf             -5.860e-02 -2.107e-01  8.704e-02 2119.751
traitexp:traitsoc.IDf              4.852e-02 -5.037e-02  1.578e-01 1410.132
traitact:traitsoc.IDf              5.360e-02 -8.365e-02  1.989e-01 2153.132
traitsoc:traitsoc.IDf              6.444e-02  5.266e-03  1.441e-01 1647.586
traitavo:traitsoc.IDf             -6.067e-02 -1.512e-01  4.456e-03 2107.655
traitale:traitsoc.IDf              9.223e-03 -6.844e-02  8.598e-02  939.829
traitoth:traitsoc.IDf              5.677e-02 -7.715e-02  1.958e-01 2305.063
traitSURVf.2:traitsoc.IDf         -2.382e+00 -8.682e+00  2.865e+00  565.215
traitREPRODf.2:traitsoc.IDf        3.130e+00 -4.928e+00  1.271e+01  357.090
traitshy:traitavo.IDf              1.408e-01 -2.867e-02  3.265e-01 1576.750
traitexp:traitavo.IDf             -7.738e-02 -2.064e-01  3.714e-02 1585.550
traitact:traitavo.IDf             -1.360e-01 -3.043e-01  2.763e-02 2203.481
traitsoc:traitavo.IDf             -6.067e-02 -1.512e-01  4.456e-03 2107.655
traitavo:traitavo.IDf              9.517e-02  7.135e-03  2.077e-01 2295.804
traitale:traitavo.IDf             -3.959e-02 -1.355e-01  4.310e-02 2194.784
traitoth:traitavo.IDf             -1.457e-01 -3.204e-01  1.578e-02 2212.509
traitSURVf.2:traitavo.IDf          1.531e+00 -4.948e+00  7.885e+00  537.643
traitREPRODf.2:traitavo.IDf       -3.644e+00 -1.376e+01  4.489e+00  512.535
traitshy:traitale.IDf             -7.758e-02 -2.636e-01  1.055e-01 1014.462
traitexp:traitale.IDf              3.528e-02 -9.630e-02  1.665e-01 1080.735
traitact:traitale.IDf              9.802e-02 -6.984e-02  2.776e-01 1574.836
traitsoc:traitale.IDf              9.223e-03 -6.844e-02  8.598e-02  939.829
traitavo:traitale.IDf             -3.959e-02 -1.355e-01  4.310e-02 2194.784
traitale:traitale.IDf              1.123e-01  1.104e-02  2.465e-01 1483.731
traitoth:traitale.IDf              9.593e-02 -6.235e-02  2.729e-01 2961.637
traitSURVf.2:traitale.IDf          6.798e-01 -6.719e+00  7.830e+00  268.147
traitREPRODf.2:traitale.IDf        3.011e+00 -7.140e+00  1.386e+01  133.107
traitshy:traitoth.IDf             -4.094e-01 -6.953e-01 -1.378e-01 2581.700
traitexp:traitoth.IDf              2.214e-01  2.541e-02  4.260e-01 3085.816
traitact:traitoth.IDf              4.051e-01  1.585e-01  6.793e-01 2619.872
traitsoc:traitoth.IDf              5.677e-02 -7.715e-02  1.958e-01 2305.063
traitavo:traitoth.IDf             -1.457e-01 -3.204e-01  1.578e-02 2212.509
traitale:traitoth.IDf              9.593e-02 -6.235e-02  2.729e-01 2961.637
traitoth:traitoth.IDf              4.509e-01  1.462e-01  7.678e-01 2232.288
traitSURVf.2:traitoth.IDf         -1.908e+00 -1.069e+01  5.693e+00  116.509
traitREPRODf.2:traitoth.IDf        7.772e+00 -8.281e+00  2.694e+01   17.087
traitshy:traitSURVf.2.IDf          3.557e+00 -3.317e+00  1.178e+01  124.945
traitexp:traitSURVf.2.IDf         -5.196e+00 -1.499e+01  1.553e+00   39.049
traitact:traitSURVf.2.IDf         -1.531e+00 -8.980e+00  5.264e+00  236.261
traitsoc:traitSURVf.2.IDf         -2.382e+00 -8.682e+00  2.865e+00  565.215
traitavo:traitSURVf.2.IDf          1.531e+00 -4.948e+00  7.885e+00  537.643
traitale:traitSURVf.2.IDf          6.798e-01 -6.719e+00  7.830e+00  268.147
traitoth:traitSURVf.2.IDf         -1.908e+00 -1.069e+01  5.693e+00  116.509
traitSURVf.2:traitSURVf.2.IDf      7.679e+02  5.534e+00  1.863e+03    2.649
traitREPRODf.2:traitSURVf.2.IDf   -5.917e+02 -1.836e+03  2.905e+01    3.133
traitshy:traitREPRODf.2.IDf       -1.479e+01 -3.523e+01  2.457e+00   11.033
traitexp:traitREPRODf.2.IDf        1.062e+01 -1.025e+00  2.238e+01   49.830
traitact:traitREPRODf.2.IDf        1.256e+01 -4.557e+00  3.235e+01   10.031
traitsoc:traitREPRODf.2.IDf        3.130e+00 -4.928e+00  1.271e+01  357.090
traitavo:traitREPRODf.2.IDf       -3.644e+00 -1.376e+01  4.489e+00  512.535
traitale:traitREPRODf.2.IDf        3.011e+00 -7.140e+00  1.386e+01  133.107
traitoth:traitREPRODf.2.IDf        7.772e+00 -8.281e+00  2.694e+01   17.087
traitSURVf.2:traitREPRODf.2.IDf   -5.917e+02 -1.836e+03  2.905e+01    3.133
traitREPRODf.2:traitREPRODf.2.IDf  1.702e+03  3.320e+01  3.092e+03    3.807

               ~idh(trait):AREAf
```

```
                     post.mean  l-95% CI  u-95% CI eff.samp
traitshy.AREAf         0.05823 6.570e-05   0.06840    25000
traitexp.AREAf         0.28311 8.641e-05   0.53534    25000
traitact.AREAf         0.04289 7.031e-05   0.07033    25000
traitsoc.AREAf         0.11718 8.381e-05   0.22016    25000
traitavo.AREAf         0.04035 6.355e-05   0.10382    25000
traitale.AREAf         0.11086 6.975e-05   0.16826    25000
traitoth.AREAf         0.17399 7.201e-05   0.23767    25000
traitSURVf.2.AREAf    59.04039 9.327e-05  47.41397    25000
traitREPRODf.2.AREAf 216.45379 7.286e-05 429.63253    24270

 R-structure:  ~us(trait):units

                                     post.mean l-95% CI u-95% CI eff.samp
traitshy:traitshy.units               0.5620600  0.33785  0.81216     3529
traitexp:traitshy.units              -0.2715002 -0.44723 -0.10257     1816
traitact:traitshy.units              -0.4911917 -0.71671 -0.28486     4776
traitsoc:traitshy.units              -0.0743685 -0.22052  0.06201     2296
traitavo:traitshy.units               0.1984121  0.04311  0.35730     1681
traitale:traitshy.units              -0.0561358 -0.21873  0.11053     1155
traitoth:traitshy.units              -0.2398584 -0.43946 -0.05436     2876
traitSURVf.2:traitshy.units           0.0060043 -0.37216  0.36431     1736
traitREPRODf.2:traitshy.units         0.0030406 -0.36034  0.37542     3625
traitshy:traitexp.units              -0.2715002 -0.44723 -0.10257     1816
traitexp:traitexp.units               0.6588662  0.46201  0.87688     3260
traitact:traitexp.units               0.1820036  0.02951  0.35068     2097
traitsoc:traitexp.units               0.0422185 -0.09914  0.17639     4354
traitavo:traitexp.units              -0.0955224 -0.24641  0.05051     4449
traitale:traitexp.units               0.0449301 -0.11387  0.19249     2498
traitoth:traitexp.units               0.0474047 -0.11180  0.21851     4285
traitSURVf.2:traitexp.units          -0.0006673 -0.38176  0.36656     2081
traitREPRODf.2:traitexp.units        -0.0003525 -0.38132  0.37239     3771
traitshy:traitact.units              -0.4911917 -0.71671 -0.28486     4776
traitexp:traitact.units               0.1820036  0.02951  0.35068     2097
traitact:traitact.units               0.5498820  0.33420  0.78078     5279
traitsoc:traitact.units               0.0398950 -0.09915  0.18233     3049
traitavo:traitact.units              -0.1369323 -0.30501  0.01064     2092
traitale:traitact.units               0.0817461 -0.07424  0.25329     1879
traitoth:traitact.units               0.2011782  0.02946  0.39792     2424
traitSURVf.2:traitact.units          -0.0027230 -0.35911  0.36065     2150
traitREPRODf.2:traitact.units        -0.0035360 -0.36676  0.34891     3882
traitshy:traitsoc.units              -0.0743685 -0.22052  0.06201     2296
traitexp:traitsoc.units               0.0422185 -0.09914  0.17639     4354
traitact:traitsoc.units               0.0398950 -0.09915  0.18233     3049
traitsoc:traitsoc.units               0.7632689  0.57379  0.97047    18363
traitavo:traitsoc.units              -0.7299801 -0.92153 -0.54885    15619
traitale:traitsoc.units              -0.0767856 -0.22786  0.07194     6090
traitoth:traitsoc.units               0.1281871 -0.02089  0.27704     7570
traitSURVf.2:traitsoc.units          -0.0317100 -0.42502  0.36471     1793
traitREPRODf.2:traitsoc.units         0.0015618 -0.41563  0.39339     3026
traitshy:traitavo.units               0.1984121  0.04311  0.35730     1681
traitexp:traitavo.units              -0.0955224 -0.24641  0.05051     4449
traitact:traitavo.units              -0.1369323 -0.30501  0.01064     2092
traitsoc:traitavo.units              -0.7299801 -0.92153 -0.54885    15619
traitavo:traitavo.units               0.8778153  0.66556  1.11593    13689
traitale:traitavo.units              -0.0704603 -0.23655  0.08490    10099
traitoth:traitavo.units              -0.3202071 -0.49495 -0.15260     5429
traitSURVf.2:traitavo.units           0.0376822 -0.40220  0.46691     1646
traitREPRODf.2:traitavo.units        -0.0022087 -0.44882  0.43015     2827
traitshy:traitale.units              -0.0561358 -0.21873  0.11053     1155
traitexp:traitale.units               0.0449301 -0.11387  0.19249     2498
traitact:traitale.units               0.0817461 -0.07424  0.25329     1879
traitsoc:traitale.units              -0.0767856 -0.22786  0.07194     6090
traitavo:traitale.units              -0.0704603 -0.23655  0.08490    10099
traitale:traitale.units               0.8707663  0.65169  1.10749     5521
traitoth:traitale.units               0.0117040 -0.15824  0.18183     5069
traitSURVf.2:traitale.units           0.0009537 -0.45012  0.43941     1848
traitREPRODf.2:traitale.units         0.0104348 -0.44781  0.44874     2871
traitshy:traitoth.units              -0.2398584 -0.43946 -0.05436     2876
traitexp:traitoth.units               0.0474047 -0.11180  0.21851     4285
traitact:traitoth.units               0.2011782  0.02946  0.39792     2424
traitsoc:traitoth.units               0.1281871 -0.02089  0.27704     7570
```

```
traitavo:traitoth.units           -0.3202071 -0.49495 -0.15260     5429
traitale:traitoth.units            0.0117040 -0.15824  0.18183     5069
traitoth:traitoth.units            0.6522748  0.41474  0.90377     2487
traitSURVf.2:traitoth.units       -0.0185928 -0.41892  0.37589     2291
traitREPRODf.2:traitoth.units     -0.0020804 -0.37409  0.38453     3546
traitshy:traitSURVf.2.units        0.0060043 -0.37216  0.36431     1736
traitexp:traitSURVf.2.units       -0.0006673 -0.38176  0.36656     2081
traitact:traitSURVf.2.units       -0.0027230 -0.35911  0.36065     2150
traitsoc:traitSURVf.2.units       -0.0317100 -0.42502  0.36471     1793
traitavo:traitSURVf.2.units        0.0376822 -0.40220  0.46691     1646
traitale:traitSURVf.2.units        0.0009537 -0.45012  0.43941     1848
traitoth:traitSURVf.2.units       -0.0185928 -0.41892  0.37589     2291
traitSURVf.2:traitSURVf.2.units    0.5000000  0.50000  0.50000        0
traitREPRODf.2:traitSURVf.2.units  0.0000000  0.00000  0.00000        0
traitshy:traitREPRODf.2.units      0.0030406 -0.36034  0.37542     3625
traitexp:traitREPRODf.2.units     -0.0003525 -0.38132  0.37239     3771
traitact:traitREPRODf.2.units     -0.0035360 -0.36676  0.34891     3882
traitsoc:traitREPRODf.2.units      0.0015618 -0.41563  0.39339     3026
traitavo:traitREPRODf.2.units     -0.0022087 -0.44882  0.43015     2827
traitale:traitREPRODf.2.units      0.0104348 -0.44781  0.44874     2871
traitoth:traitREPRODf.2.units     -0.0020804 -0.37409  0.38453     3546
traitSURVf.2:traitREPRODf.2.units  0.0000000  0.00000  0.00000        0
traitREPRODf.2:traitREPRODf.2.units  0.5000000  0.50000  0.50000      0

Location effects: cbind(shy, exp, act, soc, avo, ale, oth, SURVf, REPRODf)
~ trait - 1 + trait:YEARf + trait:bm + at.level(trait, c(1:8)):SEXf
+ at.level(trait, c(1:7)):ORDERf

                                  post.mean   l-95% CI   u-95% CI  eff.samp   pMCMC
traitshy                          1.163e+00  3.860e-01  1.947e+00  1142.963 0.00584 **
traitexp                         -1.082e+00 -1.899e+00 -2.476e-01  7206.673 0.01768 *
traitact                         -1.142e+00 -1.938e+00 -3.802e-01  1207.407 0.00584 **
traitsoc                          3.867e-01 -3.783e-01  1.152e+00 17386.903 0.30568
traitavo                         -4.133e-01 -1.210e+00  3.761e-01 15426.094 0.30160
traitale                          7.859e-01 -2.536e-02  1.620e+00 10654.590 0.05968 .
traitoth                         -1.079e+00 -1.934e+00 -2.472e-01  6120.889 0.01496 *
traitSURVf.2                      2.006e+01  2.404e+00  3.820e+01    15.840 0.00352 **
traitREPRODf.2                    5.231e+01  9.045e+00  8.762e+01     5.016 0.00464 **
traitshy:YEARf2017               -3.657e-01 -7.515e-01 -3.357e-04   594.488 0.05584 .
traitexp:YEARf2017                5.352e-01  1.967e-01  8.770e-01  1262.366 0.00176 **
traitact:YEARf2017                2.873e-01 -7.349e-02  6.612e-01   771.762 0.12584
traitsoc:YEARf2017                1.999e-01 -1.218e-01  5.228e-01  7843.611 0.22544
traitavo:YEARf2017               -1.889e-01 -5.384e-01  1.598e-01  7482.196 0.29032
traitale:YEARf2017               -1.012e+00 -1.368e+00 -6.565e-01  5093.180 < 4e-05 ***
traitoth:YEARf2017                9.940e-01  6.091e-01  1.366e+00  1932.287 < 4e-05 ***
traitSURVf.2:YEARf2017           -1.282e+01 -2.488e+01 -7.537e-01    16.274 0.00360 **
traitREPRODf.2:YEARf2017         -3.665e+01 -5.956e+01 -8.274e+00     7.730 < 4e-05 ***
traitshy:bm                      -2.331e-02 -2.209e-01  1.724e-01   423.709 0.80328
traitexp:bm                       1.074e-01 -6.690e-02  2.872e-01  1124.699 0.22864
traitact:bm                      -3.828e-02 -2.299e-01  1.564e-01   555.309 0.69840
traitsoc:bm                       4.543e-02 -1.143e-01  2.184e-01  7386.125 0.59152
traitavo:bm                      -6.584e-02 -2.410e-01  1.188e-01  9276.265 0.47200
traitale:bm                      -3.108e-02 -2.134e-01  1.548e-01  4643.310 0.74120
traitoth:bm                       9.067e-02 -1.009e-01  2.944e-01  1470.563 0.35728
traitSURVf.2:bm                   5.168e+00 -6.038e-01  1.376e+01    33.977 0.06960 .
traitREPRODf.2:bm                 3.380e+01  9.216e+00  5.585e+01     6.287 < 4e-05 ***
at.level(trait, c(1:8))1:SEXfF   -9.220e-02 -5.081e-01  3.590e-01   641.722 0.66024
at.level(trait, c(1:8))2:SEXfF   -1.123e-01 -5.049e-01  2.650e-01  1390.806 0.56376
at.level(trait, c(1:8))3:SEXfF    2.812e-01 -1.537e-01  7.004e-01   824.295 0.19976
at.level(trait, c(1:8))4:SEXfF    1.918e-02 -3.611e-01  3.757e-01  7707.227 0.91888
at.level(trait, c(1:8))5:SEXfF   -8.581e-02 -4.812e-01  3.115e-01 12057.052 0.67792
at.level(trait, c(1:8))6:SEXfF    4.603e-02 -3.559e-01  4.511e-01  5922.144 0.82168
at.level(trait, c(1:8))7:SEXfF   -7.188e-02 -5.112e-01  3.763e-01  2107.016 0.75104
at.level(trait, c(1:8))8:SEXfF    5.933e+00 -7.654e+00  2.076e+01    44.138 0.38544
at.level(trait, c(1:7))1:ORDERf1 -1.302e+00 -1.983e+00 -5.981e-01  1137.946 0.00064 ***
at.level(trait, c(1:7))2:ORDERf1  1.156e+00  4.910e-01  1.842e+00  5411.995 0.00056 ***
at.level(trait, c(1:7))3:ORDERf1  1.267e+00  5.860e-01  1.968e+00  1133.775 0.00088 ***
at.level(trait, c(1:7))4:ORDERf1 -9.238e-01 -1.589e+00 -2.037e-01 15884.551 0.00936 **
at.level(trait, c(1:7))5:ORDERf1  7.733e-01  2.161e-02  1.518e+00 15328.803 0.04192 *
at.level(trait, c(1:7))6:ORDERf1 -3.904e-01 -1.137e+00  3.699e-01 11061.712 0.31080
at.level(trait, c(1:7))7:ORDERf1  9.066e-01  1.869e-01  1.629e+00  5175.626 0.01432 *
at.level(trait, c(1:7))1:ORDERf2 -5.485e-01 -1.225e+00  1.401e-01  1660.388 0.11344
at.level(trait, c(1:7))2:ORDERf2  6.090e-01 -8.695e-02  1.261e+00  6790.527 0.07768 .
```

```
at.level(trait, c(1:7))3:ORDERf2  5.116e-01 -1.478e-01  1.210e+00  1535.643 0.13512
at.level(trait, c(1:7))4:ORDERf2 -6.174e-01 -1.330e+00  7.908e-02 18001.470 0.08512 .
at.level(trait, c(1:7))5:ORDERf2  5.807e-01 -1.816e-01  1.351e+00 15534.290 0.13584
at.level(trait, c(1:7))6:ORDERf2 -3.472e-01 -1.118e+00  4.223e-01 13443.915 0.37600
at.level(trait, c(1:7))7:ORDERf2  5.388e-01 -1.754e-01  1.252e+00  7257.387 0.13736
at.level(trait, c(1:7))1:ORDERf3 -6.775e-01 -1.362e+00  1.316e-02  6844.801 0.05280 .
at.level(trait, c(1:7))2:ORDERf3  8.257e-01  8.915e-02  1.557e+00 13999.990 0.02816 *
at.level(trait, c(1:7))3:ORDERf3  7.884e-01  9.720e-02  1.452e+00  8834.228 0.02432 *
at.level(trait, c(1:7))4:ORDERf3 -5.728e-01 -1.368e+00  1.902e-01 21141.031 0.15184
at.level(trait, c(1:7))5:ORDERf3  5.280e-01 -3.351e-01  1.352e+00 19538.430 0.21664
at.level(trait, c(1:7))6:ORDERf3 -1.111e-01 -9.648e-01  7.125e-01 18603.313 0.79704
at.level(trait, c(1:7))7:ORDERf3  7.053e-01 -5.125e-02  1.417e+00 19213.410 0.06040 .
```

Correlation analysis

correlation with survival

```
Correlation estimates for shyness-survival
      Mean: 0.1831626
      Marginal parameters posterior mode: 0.2095492
      Highest Posterior Density interval: -0.1991068 0.5522757

Correlation estimates for exploration-survival
      Mean: -0.3849028
      Marginal parameters posterior mode: -0.4722765
      Highest Posterior Density interval: -0.832006 0.08709697

Correlation estimates for activity-survival
      Mean: -0.08559423
      Marginal parameters posterior mode: -0.1089444
      Highest Posterior Density interval: -0.4744907 0.2906504

Correlation estimates for sociability-survival
      Mean: -0.3765683
      Marginal parameters posterior mode: -0.7013156
      Highest Posterior Density interval: -0.9602636 0.3722449

Correlation estimates for avoidance-survival
      Mean: 0.1932249
      Marginal parameters posterior mode: 0.3753593
      Highest Posterior Density interval: -0.5249633 0.8858688

Correlation estimates for alert-survival
      Mean: 0.1072261
      Marginal parameters posterior mode: 0.1480126
      Highest Posterior Density interval: -0.6155345 0.8529692

Correlation estimates for other-survival
      Mean: -0.1013331
      Marginal parameters posterior mode: -0.1225898
      Highest Posterior Density interval: -0.5289756 0.3397948
```

correlation with reproduction

```
Correlation estimates for shyness-reproduction
      Mean: -0.5307868
      Marginal parameters posterior mode: -0.9621812
      Highest Posterior Density interval: -0.9969874 0.01824822

Correlation estimates for exploration-reproduction
      Mean: 0.5747016
      Marginal parameters posterior mode: 0.6180477
      Highest Posterior Density interval: 0.1146062 0.9829026

Correlation estimates for activity-reproduction
      Mean: 0.4613425
      Marginal parameters posterior mode: 0.7992988
```

```
      Highest Posterior Density interval: -0.1323227 0.9889971

Correlation estimates for sociability-reproduction
      Mean: 0.3048538
      Marginal parameters posterior mode: 0.4881044
      Highest Posterior Density interval: -0.450145 0.920923

Correlation estimates for avoidance-reproduction
      Mean: -0.3035649
      Marginal parameters posterior mode: -0.3901898
      Highest Posterior Density interval: -0.9253046 0.357138

Correlation estimates for alert-reproduction
      Mean: 0.190059
      Marginal parameters posterior mode: 0.3306692
      Highest Posterior Density interval: -0.6079487 0.916729

Correlation estimates for other-reproduction
      Mean: 0.3300943
      Marginal parameters posterior mode: 0.1276445
      Highest Posterior Density interval: -0.2284801 0.9731371
```

3. **Differences between Red-Grey and Red-only areas**

```
-----------------------------------------------

Iterations = 50001:1049961
Thinning interval = 40
Number of chains = 1
Sample size per chain = 25000

      differences between areas for shyness and survival
          Mean             SD        Naive SE Time-series SE
     -0.134444       0.260377       0.001647       0.017764
 Quantiles for each variable:
    2.5%      25%      50%      75%    97.5%
-0.63786 -0.31287 -0.13533  0.04219  0.38445

      differences between areas for exploration and survival
          Mean             SD        Naive SE Time-series SE
      0.191647       0.439804       0.002782       0.030417
Quantiles for each variable:
   2.5%     25%     50%     75%   97.5%
-0.6319 -0.1200  0.1761  0.4984  1.0652


      differences between areas for activity and survival
          Mean             SD        Naive SE Time-series SE
     -0.033527       0.278090       0.001759       0.020115
Quantiles for each variable:
    2.5%      25%      50%      75%    97.5%
-0.55900 -0.22549 -0.03935  0.15418  0.52301

      differences between areas for sociability and survival
          Mean             SD        Naive SE Time-series SE
      0.314884       0.422907       0.002675       0.015759
Quantiles for each variable:
    2.5%      25%      50%      75%    97.5%
-0.61575  0.04598  0.35763  0.62009  1.02673

      differences between areas for avoidance and survival
          Mean             SD        Naive SE Time-series SE
     -0.155172       0.430969       0.002726       0.020038
Quantiles for each variable:
   2.5%     25%     50%     75%   97.5%
-0.9406 -0.4669 -0.1704  0.1378  0.7237
```

```
      differences between areas for alert and survival
          Mean             SD       Naive SE Time-series SE
     -0.195212       0.519388       0.003285       0.025106
Quantiles for each variable:
   2.5%     25%     50%     75%   97.5%
-1.1718 -0.5589 -0.2095  0.1625  0.8360

      differences between areas for other and survival
          Mean             SD       Naive SE Time-series SE
     -0.107983       0.325613       0.002059       0.031546
Quantiles for each variable:
   2.5%     25%     50%     75%   97.5%
-0.7169 -0.3355 -0.1182  0.1112  0.5510

      differences between areas for shyness and reproduction
          Mean             SD       Naive SE Time-series SE
      0.596056       0.466259       0.002949       0.101868
Quantiles for each variable:
   2.5%     25%     50%     75%   97.5%
-0.3528  0.2900  0.5768  0.9279  1.4546

      differences between areas for exploration and reproduction
          Mean             SD       Naive SE Time-series SE
     -0.808367       0.483519       0.003058       0.048845
Quantiles for each variable:
   2.5%     25%     50%     75%   97.5%
-1.6404 -1.1740 -0.8371 -0.4739  0.1819

      differences between areas for activity and reproduction
          Mean             SD       Naive SE Time-series SE
     -0.550726       0.469683       0.002971       0.103216
Quantiles for each variable:
   2.5%     25%     50%     75%   97.5%
-1.4052 -0.8821 -0.5527 -0.2493  0.4452

      differences between areas for sociability and reproduction
          Mean             SD       Naive SE Time-series SE
      -0.60127        0.43805        0.00277        0.02059
Quantiles for each variable:
   2.5%     25%     50%     75%   97.5%
-1.3863 -0.9142 -0.6244 -0.3078  0.3114

      differences between areas for avoidance and reproduction
          Mean             SD       Naive SE Time-series SE
      0.565032       0.449247       0.002841       0.030783
Quantiles for each variable:
   2.5%     25%     50%     75%   97.5%
-0.3661  0.2633  0.5852  0.8892  1.3653

      differences between areas for alert and reproduction
          Mean             SD       Naive SE Time-series SE
     -0.114443       0.611074       0.003865       0.053770
Quantiles for each variable:
   2.5%     25%     50%     75%   97.5%
-1.2518 -0.5622 -0.1235  0.3200  1.0877

      differences between areas for other and reproduction
          Mean             SD       Naive SE Time-series SE
     -0.498838       0.492757       0.003116       0.111526
Quantiles for each variable:
   2.5%     25%     50%     75%   97.5%
-1.4371 -0.8622 -0.4516 -0.1489  0.4021
```
